# Serine protease HtrA promotes *Campylobacter jejuni* intestinal colonization through degrading antimicrobial peptide LL-37

**DOI:** 10.1126/sciadv.aee1996

**Published:** 2026-05-20

**Authors:** Xiaofei Li, Mengjie Zhang, Zhenzhen Xu, Yunlu Li, Su Bian, Yuanyue Tang, Xinan Jiao, Ozan Gundogdu, Jinlin Huang

**Affiliations:** ^1^ Jiangsu Key Laboratory of Zoonosis, Jiangsu Co-Innovation Center for Prevention and Control of Important Animal Infectious Diseases and Zoonoses, Yangzhou University, Yangzhou, Jiangsu 225009, China.; ^2^ School of Life and Health Sciences, Hainan Province Key Laboratory of One Health, Collaborative Innovation Center of Life and Health, Hainan University, Haikou, Hainan 570228, China.; ^3^ Key Laboratory of Prevention and Control of Biological Hazard Factors (Animal Origin) for Agrifood Safety and Quality, Ministry of Agriculture of China, Yangzhou, Jiangsu 225009, China.; ^4^ Joint International Research Laboratory of Agriculture and Agri-product Safety, Ministry of Education of China, Yangzhou, Jiangsu 225009, China.; ^5^ Department of Infection Biology, Faculty of Infectious and Tropical Diseases, London School of Hygiene & Tropical Medicine, Keppel Street, London WC1E 7HT, UK.; ^6^ College of Life Sciences, Hainan Normal University, Haikou 571158, China.

## Abstract

*Campylobacter jejuni* (*C. jejuni*) is a leading cause of human gastroenteritis worldwide and must overcome intestinal innate immunity, including antimicrobial peptide LL-37. However, how *C. jejuni* responds to LL-37 remains unclear. Here, we showed that *C. jejuni* infection stimulates intestinal epithelial cells to secrete LL-37, exhibiting effective antibacterial activity against 86.3% of *C. jejuni* clinical isolates by disrupting essential processes required for bacterial survival. A subset of isolates displays intrinsic resistance, enabling successful intestinal colonization. We further identified conserved serine protease HtrA as the key determinant of resistance. Mechanistically, LL-37 exposure activates transcriptional regulator NssR, which up-regulates *htrA* expression. Secreted HtrA cleaves LL-37 at Ile^20^-Val^21^ site, abolishing its antimicrobial activity and promoting bacterial survival. In light of this mechanism, we developed a noncleavable LL-37^I20M/V21R^ that displays enhanced antibacterial activity and promotes bacterial clearance in mice. Together, our findings uncover mechanistic insights into interactions between human enteric pathogens and antimicrobial peptides and provide a potential strategy for combating *C. jejuni* infection.

## Teaser

HtrA enables *C. jejuni* to evade LL-37 killing by cleaving it, revealing a therapeutic target and a noncleavable LL-37 analog.

## INTRODUCTION

*Campylobacter jejuni* (*C. jejuni*) is a leading cause of bacterial gastroenteritis worldwide, responsible for millions of cases of diarrheal disease each year (*1*–*3*). In addition to the acute symptoms of campylobacteriosis, which include bloody diarrhea, fever, and abdominal pain, *C. jejuni* infection can also lead to severe and potentially life-threatening consequences such as Guillain-Barré syndrome, Miller-Fisher syndrome, and colorectal cancer (*4*, *5*). *C. jejuni* is a prevalent commensal bacterium in the intestinal tracts of chickens and other livestock (*6*), and consumption of contaminated animal products is the primary route of human infection (*7*). Upon entry into the intestinal tract, *C. jejuni* encounters both host innate and adaptive immune defenses that act to restrict bacterial growth (*8*). To establish successful colonization, *C. jejuni* must therefore overcome multiple host defense mechanisms, including antimicrobial peptides (AMPs) (*9*).

AMPs, including defensins and cathelicidin, are key components of innate immune system and constitute the first line of defense against invading pathogens in diverse organisms (*10*–*12*). It has previously been shown that *C. jejuni* infection induces the expression of human β-defensins 2 and 3 in intestinal epithelial cells. These defensins exhibit potent antibacterial activity against *C. jejuni* in vitro (*9*), suggesting that they may contribute to controlling its colonization and survival in the intestine. In contrast, the involvement of cathelicidin in the host response to *C. jejuni* remains unclear. The AMP LL-37 is the only known member of the cathelicidin family peptides found in humans and primarily expressed by neutrophils, epithelial cells, and keratinocytes (*13*). It has attracted considerable attention due to its broad-spectrum antibacterial activity against both Gram-negative and Gram-positive bacteria, including *Pseudomonas*, *Escherichia*, *Staphylococcus*, and *Enterococcus* genera (*13*–*17*). As an α-helical amphipathic peptide, LL-37 exerts its direct antibacterial activity primarily through interactions with bacterial membranes (*18*–*20*), or by penetrating the inner membrane to target intracellular molecules, including DNA and proteins (*16*, *21*).

Through prolonged coevolution with AMPs, bacteria have in turn developed diverse strategies to counteract the antibacterial activity of LL-37, thereby enhancing their ability to survive and establish infection (*22*–*24*). The most common resistance mechanisms include modifications of cell surface structures that reduce LL-37 binding, activation of efflux pumps that expel the peptide, and secretion of proteases that degrade LL-37 (*11*, *15*, *25*). Notably, in many enteric bacteria such as *Salmonella enterica*, *Helicobacter pylori*, and *Enterococcus faecalis* (*22*, *23*, *26*), resistance to LL-37 is predominantly achieved through alterations in the composition or charge of the cell membrane. However, whether and how *C. jejuni*, a major enteric pathogen responsible for severe diarrheal disease, responds to LL-37 during intestinal colonization remains unclear.

In this study, we demonstrate that epithelial LL-37 production is up-regulated upon *C. jejuni* infection and effectively kills most clinical isolates by binding bacterial DNA and disrupting physiological processes. Intriguingly, we identify a subset of *C. jejuni* isolates that exhibit resistance to LL-37, enabling them to successfully colonize the intestinal tract. These isolates evade LL-37 killing through a specific mechanism involving the serine protease HtrA, whose expression is directly activated by the transcriptional regulator NssR in response to LL-37. HtrA specifically cleaves LL-37 at the Ile^20^-Val^21^ site, thereby abolishing its antibacterial activity. Furthermore, we engineered a noncleavable LL-37^I20M/V21R^ with enhanced antibacterial efficacy that markedly improves bacterial clearance in vivo. Our study provides a mechanistic insight into how *C. jejuni* resists the bactericidal activity of LL-37 and develops LL-37^I20M/V21R^ as a promising candidate against *C. jejuni* infections.

## RESULTS

### LL-37 exerts potent antibacterial activity against *C. jejuni* clinical isolates

**Fig. 1.**
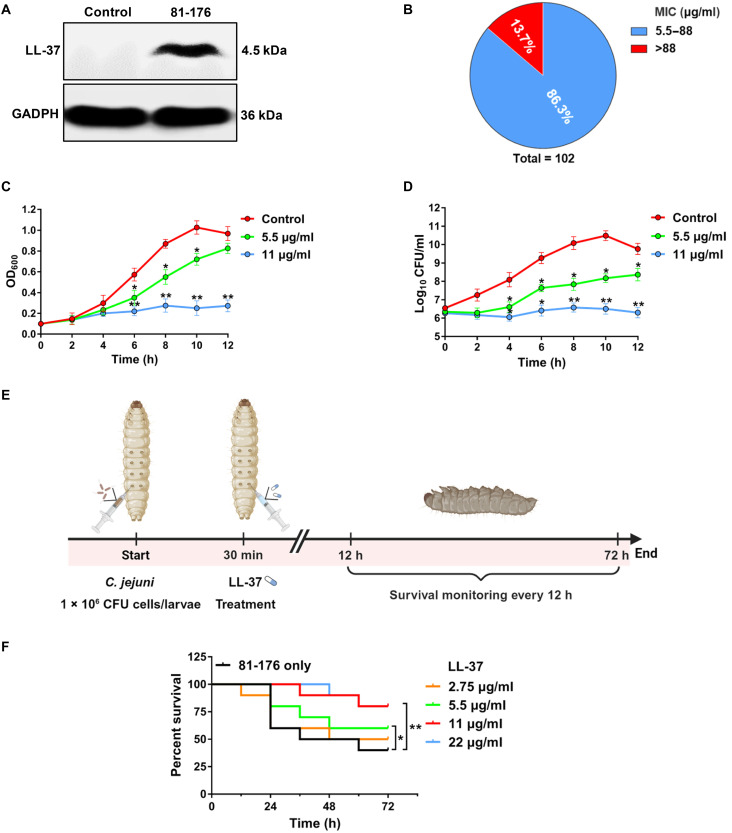
*C. jejuni* induces Caco-2 cells to express LL-37 to elicit bacteriostatic activity. (**A**) WB of LL-37 in cell extracts of Caco-2 cells infected with or without *C. jejuni* 81-176 strain at an MOI of 20:1 for 24 hours (h). GADPH (glyceraldehyde-3-phosphate dehydrogenase) was used as a loading control. (**B**) Pie chart indicating the MIC of LL-37 against 102 clinical isolates of *C. jejuni*. Blue indicates MIC values between 5.5 and 88 μg/ml, while red represents values exceeding 88 μg/ml. (**C** and **D**) Bacterial growth kinetics of *C. jejuni* 81-176 treated with or without LL-37 at 1 × MIC (5.5 μg/ml) and 2 × MIC (11 μg/ml). The growth curves were measured as OD_600_ (C) and bacterial counts (log_10_ CFU/ml) (D). (**E**) Schematic of the in vivo experiments. *G. mellonella* larvae were injected with *C. jejuni* 81-176 strain (10^6^ bacterial cells per larvae) into the last left proleg. Thirty minutes after infection, the larvae were treated with LL-37 at various concentrations via injection into the last right proleg. Larval survival was monitored over the following 72 hours. Created in BioRender. Li, X. (2026) https://BioRender.com/izqtrbq. (**F**) Survival curves of *G. mellonella* larvae infected with *C. jejuni* 81-176 and treated with LL-37 over 72 hours. Statistical analysis of survival curves used the log-rank test. All data [(C), (D), and (F)] are means ± SD of three independent experiments. Significance was determined by Student’s *t* test. *P < 0.05, **P < 0.01.

To determine whether *C. jejuni* infection induces LL-37 expression in intestinal epithelial cells, we used human colon epithelial Caco-2 cells as a model and infected them with *C. jejuni* 81-176 strain at a multiplicity of infection (MOI) of 20. After 24 hours of incubation, total cellular proteins were extracted and analyzed by Western blot (WB). As shown in Fig. 1A, *C. jejuni* infection resulted in a strong induction of LL-37 expression in Caco-2 cells compared with noninfected controls, suggesting that intestinal epithelial cells may up-regulate LL-37 expression as a host defense mechanism to eliminate *C. jejuni*. To investigate whether LL-37 exerts bactericidal activity against *C. jejuni*, we initially evaluated the susceptibility of 102 clinical isolates to LL-37 by determining their minimum inhibitory concentrations (MICs) in vitro (table S1). The results showed that 88 isolates (86.3%) displayed MIC values ranging from 5.5 to 88 μg/ml, whereas the remaining 14 isolates (13.7%) showed MIC values exceeding 88 μg/ml, indicating that LL-37 exerted broad-spectrum antibacterial activity against *C. jejuni* (Fig. 1B). For subsequent analysis, isolates with MIC values ≤88 μg/ml were defined as LL-37–sensitive isolates, while those with MIC values >88 μg/ml were defined as LL-37–resistant isolates. The most prevalent clonal complexes among the LL-37–resistant isolates were CC-21 and CC-353, accounting for 42.8% (6 of 14) (fig. S1A and table S1).

To further validate the antibacterial efficacy of LL-37 against *C. jejuni*, we selected the reference strain 81-176 as a representative of LL-37–sensitive strains, with an MIC of 11 μg/ml and a minimum bactericidal concentration (MBC) of 22 μg/ml (table S2). The control scrambled LL-37 (Scrm) showed no detectable antibacterial activity against 81-176 strain, even at concentrations up to 88 μg/ml (table S2), supporting the critical role of the native LL-37 in exerting its antibacterial effect. Bacterial growth in liquid culture was subsequently monitored over time in the presence of LL-37 at 5.5 μg/ml (one-half MIC) and 11 μg/ml (MIC). *C. jejuni* cells exhibited growth inhibition when exposed to LL-37 at 11 μg/ml (Fig. 1C). This resulted in a significant reduction (~1000-fold) in bacterial colony counts compared to the untreated control (*P* < 0.01; Fig. 1D).

Next, we assessed the potential cytotoxicity exhibited by LL-37 in Caco-2 cells. Across varying concentrations (0 to 88 μg/ml), LL-37 exhibited a negligible effect on cell viability (less than 8%) (fig. S1B), suggesting the excellent cell compatibility and safety of LL-37. Consequently, the antibacterial efficiency of LL-37 in a safe concentration range was further evaluated in vivo using a *Galleria mellonella* (*G. mellonella*) infection model. Larvae were infected with *C. jejuni* 81-176 strain [1 × 10^6^ colony-forming unit (CFU)], followed by LL-37 (2.75 to 22 μg/ml, corresponding to 1/4-2 MIC) treatment 30 min after infection, and their survival was recorded over a 72-hour period (Fig. 1E). We found that treatment with LL-37 significantly increased the survival time and reduced infection-induced mortality (Fig. 1F), indicating that LL-37 can protect host from *C. jejuni* infection. Collectively, these findings confirm that LL-37 exerts potent antibacterial activity against *C. jejuni* both in vitro and in vivo.

### LL-37 induces *C. jejuni* death via directly targeting genomic DNA

**Fig. 2.**
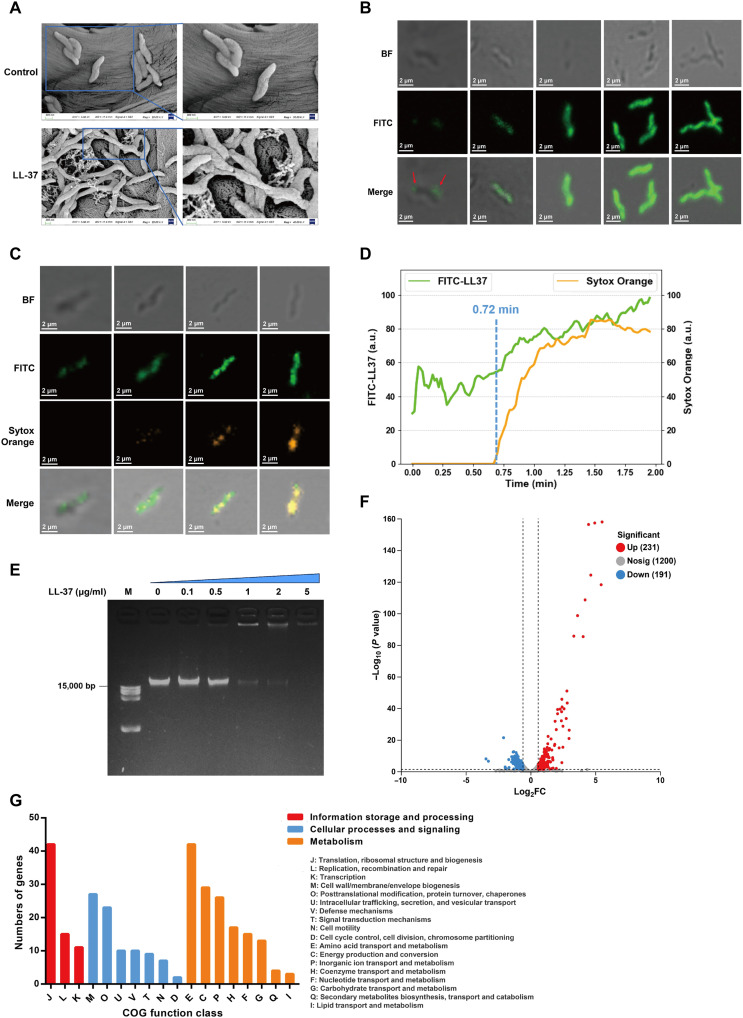
The mechanism of action of LL-37 against *C. jejuni*. (**A**) SEM images of *C. jejuni* 81-176 strain treated with or without LL-37 (2 × MIC) for 2 hours. The right panels present enlarged views of the regions indicated by boxes in the corresponding left panels. (**B**) Confocal laser scanning microscopy (CLSM) images depicting the distribution of LL-37 after entrance into *C. jejuni* 81-176 cells. Green fluorescence represents FITC-labeled LL-37, the red arrows represent the location of FITC-labeled LL-37. From left to right, the columns show representative *C. jejuni* cells at different stages of interaction with FITC-labeled LL-37, ranging from initial surface attachment to substantial intracellular accumulation. Scale bar, 2 μm. (**C**) CLSM analysis of membrane permeability changes in *C. jejuni* 81-176 following LL-37 treatment. Green fluorescence represents FITC-labeled LL-37, orange fluorescence Sytox Orange stains nuclei. From left to right, the columns show representative *C. jejuni* cells at different stages of membrane permeabilization after LL-37 treatment, ranging from intact membranes to fully permeabilized membranes. Scale bar, 2 μm. (**D**) FITC-labeled LL-37 and Sytox Orange intensity versus time in real-time observations of *C. jejuni* 81-176 cells treated with LL-37 (MIC). The green line represents FITC-labeled LL-37 and the orange line represents Sytox Orange. (**E**) Gel retardation assay. The interaction of LL-37 with *C. jejuni* 81-176 genomic DNA was assessed by gel retardation. LL-37 concentrations in each lane increase serially from 0.1 to 5 μg/ml. M, DL15000 DNA marker. (**F**) Volcano plot depicting differentially expressed genes in *C. jejuni* 81-176 strain exposed to LL-37 (5.5 μg/ml). Red dots represent significantly up-regulated genes, while blue dots represent significantly down-regulated genes. The *y* axis denotes − log_10_ (*P* value), while the *x* axis shows log_2_FC values. (**G**) RNA-seq results displayed by clusters of orthologous groups (COG) functional categories. Genes with functions that fall within two different groups are represented within both of the groups. a.u., arbitrary units.

The common mechanism of action of AMPs involves bacterial lysis through the formation of pores in the bacterial membranes (*27*). To determine whether LL-37 has effects on *C. jejuni* membrane, the 81-176 strain was incubated with LL-37, and potential morphological changes of membrane were examined by scanning electron microscopy (SEM). As shown in Fig. 2A, the control cells displayed normal and smooth surface structures. In contrast, a treatment of LL-37 resulted in clear morphological changes, including surface roughening, folding, and occasional membrane rupture (Fig. 2A). We further found that treatment with LL-37 (22 μg/ml) led to leakage of both nucleic acids and proteins leakage from *C. jejuni* cells, indicating that LL-37 may disrupt membrane integrity and cause structural damage to *C. jejuni* (fig. S2, A and B).

To more comprehensively see the behaviors of LL-37 on cell membrane and inside the bacterium, we incubated *C. jejuni* 81-176 cells with fluorescein isothiocyanate (FITC)–labeled LL-37. Confocal fluorescence images showed that FITC-labeled LL-37 initially attached to the membrane, followed by substantial intracellular accumulation (Fig. 2B). We next used the nucleic acid stain SYTOX Orange to estimate the permeability of membrane. The results showed that SYTOX Orange fluorescence was initially undetectable upon LL-37 binding to the membrane (Fig. 2C). As LL-37 progressively accumulated inside the cell, SYTOX Orange fluorescence became apparent and rapidly diffused throughout the cytoplasm (Fig. 2C), indicating a gradual increase in membrane permeability. To further resolve the dynamics of LL-37 entry, we tracked the entry of LL-37 into single live *C. jejuni* cells in real time by super-resolution microscopy. We observed that LL-37 exhibited rapid binding and penetration activities against *C. jejuni* (within 2 min) in a time-dependent manner (movie S1). At *t* = 0.72 min, the membrane is permeabilized and SYTOX Orange rapidly gains access to the cytoplasmic space (movie S1 and Fig. 2D). These findings indicate that LL-37 rapidly penetrates membrane and accumulates intracellularly, ultimately leading to membrane disruption in *C. jejuni*.

The aforementioned results indicate that LL-37 acts as a cell-penetrating AMP against *C. jejuni*, which typically interacts with cytosolic targets such as nucleic acids (*13*). Therefore, to determine whether LL-37 can interact with *C. jejuni* 81-176 genomic DNA, a gel retardation assay was performed. As a positive control, LL-37 bound to *Escherichia coli* (*E. coli*) genomic DNA in a dose-dependent manner (fig. S2C), which is consistent with previous studies (*16*, *28*). Similarly, we observed that increasing concentrations of LL-37 resulted in a gradual retardation of *C. jejuni* genomic DNA migration (Fig. 2E). This finding led us to hypothesize that the strong interaction between LL-37 and genomic DNA may inhibit essential cellular processes, such as DNA replication, transcription, and protein synthesis. To confirm our hypothesis, we conducted a transcriptome analysis of *C. jejuni* 81-176 strain following exposure to LL-37. RNA sequencing (RNA-seq) data revealed that LL-37 treatment group had 231 up-regulated and 191 down-regulated differentially expressed genes compared to the control group (Fig. 2F). In agreement, a similar result was obtained when we measured the expression of 2 randomly selected genes by quantitative reverse transcription polymerase chain reaction (qRT-PCR) (fig. S2D), confirming the reliability of the transcriptomic analysis. Further, Clusters of Orthologous Groups (COG) functional analysis showed that the down-regulated genes were primarily involved in DNA replication, transcription, translation, ribosome structure, and membrane biosynthesis (Fig. 2G and table S3). Together, these findings suggest that LL-37 penetrates the bacterial membrane and binds to genomic DNA, which interferes with essential cellular processes such as DNA replication and membrane synthesis, ultimately compromising membrane integrity and leading to the death of *C. jejuni*.

### HtrA confers LL-37 resistance to facilitate *C. jejuni* intestinal colonization

**Fig. 3.**
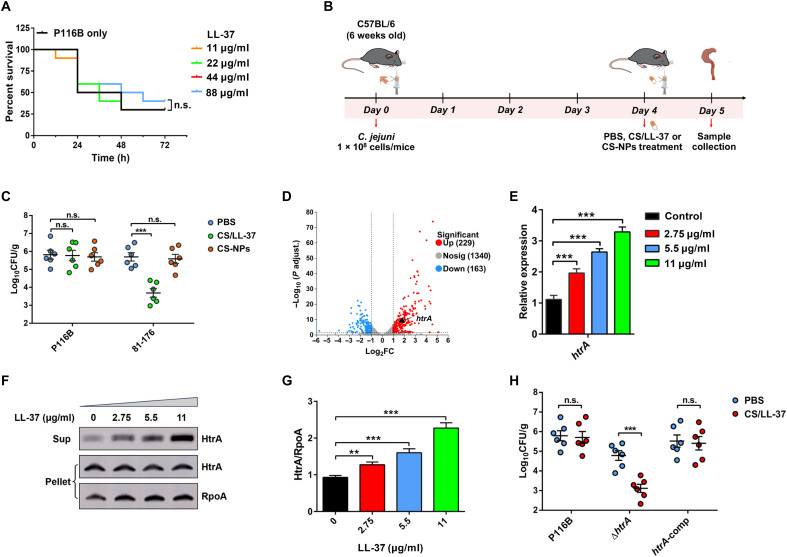
HtrA contributes to *C. jejuni* intestinal colonization through resisting LL-37. (**A**) Survival curves of *G. mellonella* larvae infected with *C. jejuni* P116B and treated with LL-37 over 72 hours. (**B**) Schematic of the in vivo experiments. C57BL/6 mice were orally infected with *C. jejuni* (10^8^ bacterial cells per mouse) on day 0. Four days postinfection, the mice were treated with PBS, CS/LL-37-NPs, or CS-NPs. Mice were euthanized on day 5, and cecum contents were collected for bacterial enumeration. Syringe illustration credit: G. D’ottavio, Scidraw.io. Created in BioRender. Li, X. (2026) https://BioRender.com/r49s6mw. (**C**) Bacterial counts in cecum contents of orally *C. jejuni*–infected mice treated with PBS (Control), CS/LL-37-NPs (88 μg/ml for P116B or 11 μg/ml for 81-176), or CS-NPs. Data are means ± SEM from three independent repeats (*n* = 6 mice per group). Bars represent mean CFU. Statistical significance was analyzed using two-way analysis of variance (ANOVA) with multiple comparisons: ***P < 0.001; n.s., not significant. (**D**) Volcano plot depicting differentially expressed genes in P116B exposed to LL-37 (11 μg/ml). Red and blue dots represent significantly up- and down-regulated genes, respectively. *y* axis: − log_10_ (*P* adjust), *x* axis: log_2_FC. (**E**) qRT-PCR analysis of *htrA* in P116B exposed to LL-37 (2.75 to 11 μg/ml). Error bars, SD of replicates. Data were analyzed using Student’s *t* test (***P < 0.001). (**F** and **G**) WB (F) and densitometry analysis (G) of HtrA in P116B strain supernatants and pellets with LL-37 (0 to 11 μg/ml). RpoA, loading control. Error bars, SD of replicates. Data were analyzed using Student’s *t* test (**P < 0.01, ***P < 0.001). (**H**) Bacterial counts in cecum contents of the orally *C. jejuni*–infected mice treated with PBS (Control) or CS/LL-37-NPs (88 μg/ml). Data are means ± SEM from three independent experiments (*n* = 6 mice per group). Bars show mean CFU. Statistical significance was determined by two-way ANOVA with multiple comparisons: ***P < 0.001; n.s., not significant.

Our results described above clearly showed that LL-37 has effective antibacterial activity against most *C. jejuni* clinical isolates. Nevertheless, certain isolates belonging to CC-21 clone complex, the most prevalent CCs among human-derived isolates (*29*), are resistant to LL-37 killing. This led us to hypothesize that these isolates may evade LL-37 defenses, thereby facilitating their survival and successful colonization of the intestinal tract. To this end, we selected an LL-37–resistant isolate, P116B, as a representative strain and used a *G. mellonella* infection model to evaluate whether resistance to LL-37 promotes bacterial survival in vivo (Fig. 1F). Approximately 25% of larvae survived after 72 hours when infected with P116B strain, but treatment with LL-37 at concentrations up to 88 μg/ml did not significantly improve larval survival, indicating that the LL-37–resistant isolate is capable of resisting LL-37 killing in vivo (Fig. 3A).

To further investigate whether *C. jejuni* P116B strain could counteract LL-37 and establish successful intestinal colonization in vivo, we used the mouse infection model. Six-week-old C57BL/6 mice were orally infected with 1 × 10^8^ viable P116B (LL-37–resistant) or 81-176 (LL-37–sensitive) strain on day 0. Four days postinfection, each mouse was orally given LL-37, and the cecal contents were sampled after treatment to counter bacterial loads (Fig. 3B). Considering the inherent instability and poor oral bioavailability of peptides, we developed a chitosan-based delivery system for LL-37 using the ionotropic gelation method (*30*). SEM analysis revealed that LL-37–loaded chitosan nanoparticles (CS/LL-37–NPs) were uniformly spherical with a homogeneous size distribution (fig. S3A). The encapsulation efficiency of LL-37 in this delivery system was high, with a rate of 89.38% (table S4). The CS/LL-37-NPs exhibited excellent pH stability across a broad pH range (2 to 10), indicating that the chitosan–tripolyphosphate (TPP) matrix effectively protects LL-37 from degradation in the intestinal environment (fig. S3B). Furthermore, time-killing kinetic assays revealed that CS/LL-37-NPs were able to cause a rapid ~3.3 log_10_ decrease in the colony counts of 81-176 strain within 2 hours and maintained a sustained antibacterial effect over the subsequent 10 hours at 1 × MIC in vitro (fig. S3C). When challenged with LL-37 in vivo, the 81-176 strain exhibited a significant reduction (~155-fold) in cecal colonization compared to the controls without LL-37 treatment (Fig. 3C). In contrast, LL-37 treatment did not significantly affect the cecal colonization of the P116B strain, which displayed a high level of bacterial loads (Fig. 3C). These findings suggest that LL-37–resistant strains have evolved specific mechanisms to evade or counteract the antimicrobial effects of LL-37, thereby facilitating intestinal colonization.

Therefore, we next sought to investigate the mechanisms underlying *C. jejuni* resistance to LL-37. To identify genes involved in the response to LL-37, a global transcriptomic analysis was performed on the P116B strain treated with or without LL-37. RNA-seq data revealed that 392 genes were differentially expressed, with 229 up-regulated and 163 down-regulated (fold change ≤0.5 and ≥2; *P* < 0.05) (Fig. 3D and table S5). KEGG pathway enrichment analysis identified P116B_01216 (*htrA*), which encodes a serine protease, as the only significantly up-regulated protease associated with cationic antibacterial peptide (CAMP) resistance (table S6). These results led us to hypothesize that HtrA might play a crucial role in mediating LL-37 resistance. To test this, we assessed the expression of *htrA* under LL-37 treatment. Both qRT-PCR and WB analyses confirmed a dose-dependent up-regulation of *htrA* expression at the transcriptional and protein levels in response to LL-37 (Fig. 3, E to G). We next generated both *htrA* deletion and complementary strains of P116B and determined their MIC against LL-37. The Δ*htrA* mutant exhibited a markedly reduced MIC compared to the wild-type (WT) strain, while complementation of *htrA* restored LL-37 resistance to levels comparable to those of the WT (table S7). Meanwhile, growth curve showed that the loss of *htrA* did not affect bacterial growth, suggesting that the observed decrease in MIC was not due to a general growth defect (fig. S3D). To explore the role of HtrA in vivo, we enumerated the bacterial loads in the cecal contents of mice infected with WT, Δ*htrA*, or *htrA*-comp strains, followed by treatment with or without LL-37. In the absence of LL-37 treatment, mice infected with Δ*htrA* mutant had fewer bacteria in cecal contents (~12-fold) than the mice infected with the WT (Fig. 3H). This is consistent with the previous study reporting that HtrA is essential for *C. jejuni* invasion of epithelial cells (*31*). We observed that when challenged with LL-37, Δ*htrA* mutant exhibited an approximately 183-fold reduction in cecal colonization relative to the WT strain (Fig. 3H), which was significantly higher than the fold decrease (~12-fold) observed in the absence of LL-37 treatment. Complementation of a functional *htrA* gene restored the colonization capacity of Δ*htrA* mutant to WT levels. Collectively, these results indicate that HtrA contributes to the ability of *C. jejuni* to counteract LL-37 killing and persist in the intestinal tract.

### HtrA specifically cleaves LL-37 between Ile^20^ and Val^21^ site

**Fig. 4.**
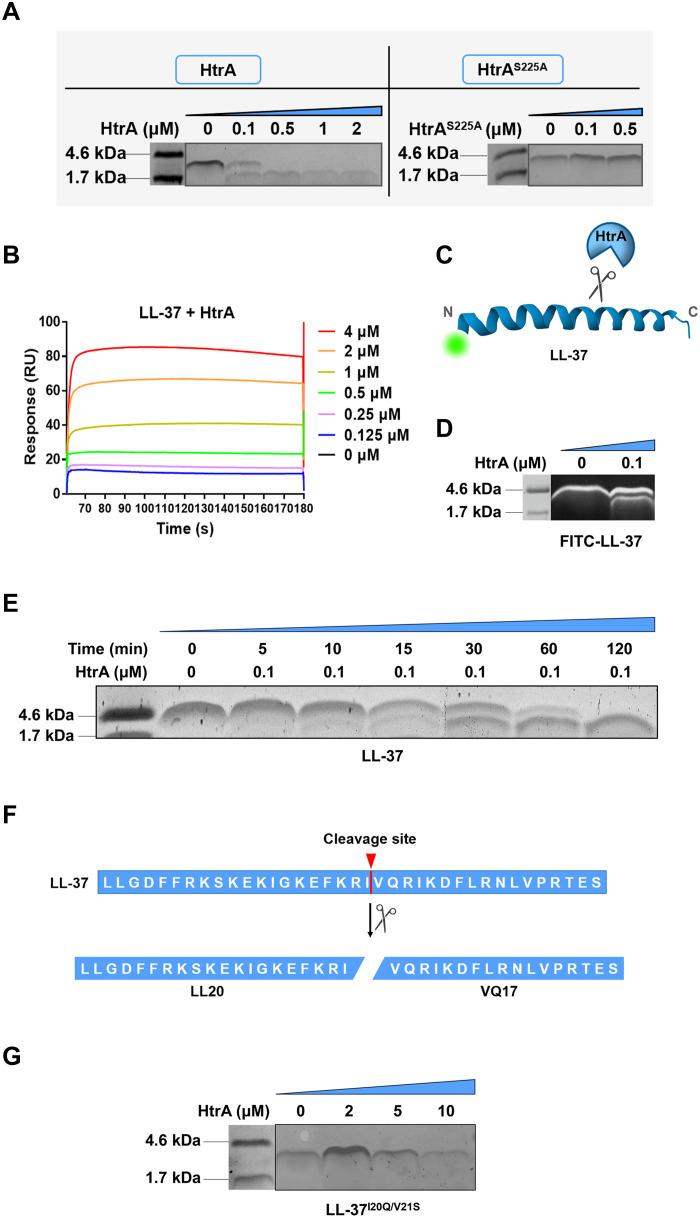
HtrA specially cleaves LL-37 between Ile^20^ and Val^21^ site. (**A**) SDS–polyacrylamide gel electrophoresis (SDS-PAGE) analysis of LL-37 cleavage after incubation with HtrA or HtrA^S225A^ for 2 hours. The gel was visualized using Coomassie blue staining. (**B**) Kinetic analysis of HtrA–LL-37 binding using SPR. The sensorgram quantifies binding of rHis-HtrA to immobilized LL-37, measured in relative response units (RUs) after background subtraction. The concentrations of rHis-HtrA applied are listed sequentially on the graphs. Affinity modeling (1:1) was used to calculate the *K*_d_ for the interaction. LL-37-HtrA: *K*_d_ = 7.362 × 10^−6^ M. (**C**) Schematic representation of HtrA cleavage pattern on FITC-labeled LL-37. (**D**) SDS-PAGE analysis of FITC-labeled LL-37 cleavage after incubation with HtrA for 2 hours. (**E**) SDS-PAGE analysis of LL-37 cleavage after incubation with HtrA for the indicated times (mins). (**F**) Diagram of the cleavage site for HtrA on LL-37. The cleavage site involves the two hydrophobic amino acids, IIe20, and Val21. LL20 (LLGDFFRKSKEKIGKEFKRI) and VQ17 (VQRIKDFLRNLVPRTES) are the peptides with the highest confidence scores based on HPLC-MS/MS results (table S8). (**G**) SDS-PAGE analysis of LL-37^I20Q/V21S^ cleavage after incubation with HtrA for 2 hours. Created in BioRender. Li, X. (2026) https://BioRender.com/aoe71fm.

Previous study has shown that LL-37 is generated from the precursor hCAP-18 through proteolytic cleavage by host-derived serine proteases (*32*). Given that *htrA* encodes a bacterial serine protease, we hypothesized that HtrA may contribute to *C. jejuni* defense against LL-37 by directly cleaving the peptide. To test this, we cloned and purified recombinant WT HtrA from P116B strain and its proteolytically inactive mutant HtrA^S225A^ (*31*) and then incubated them with LL-37 for 30 min. As shown in Fig. 4A, HtrA cleaved LL-37 at a concentration of 0.1 μM, generating a product smaller than 4.5 kDa, and completely degraded LL-37 when the concentration reached 0.5 μM. In contrast, HtrA^S225A^ mutant exhibited no detectable cleavage activity at a high concentration (2 μM). In addition, HtrA failed to cleave CRAMP, a murine cathelicidin that shares about 62% amino acid similarity with LL-37 (*15*), even at a concentration of 50 μM (fig. S4A). These results strongly suggest that HtrA is capable of specifically cleaving LL-37. Further analysis using surface plasmon resonance (SPR) demonstrated that HtrA directly binds to LL-37, with a dissociation constant (*K*_d_) of 7.362 × 10^−6^ M and a maximum response unit of 85.41, confirming a stable interaction between them (Fig. 4B). Overall, these findings suggest that HtrA specifically interacts with and cleaves LL-37.

To identify the specific cleavage site of HtrA on LL-37, we initially incubated an N-terminally FITC-labeled LL-37 with HtrA protein (Fig. 4C). The cleavage resulted in a product slightly smaller than the full-length LL-37, suggesting that HtrA cleaves LL-37 close to the C terminus (Fig. 4D). Notably, the size of the cleavage products remained consistent even after prolonged incubation with nonlabeled LL-37, supporting the notion that HtrA has a specific and unique cleavage site on LL-37 (Fig. 4E). Subsequently, we subjected the cleaved LL-37 solution to high-performance liquid chromatography–tandem mass spectrometry (HPLC-MS/MS). A total of 74 peptide fragments were identified, with the highest scoring peptides being LLGDFFRKSKEKIGKEFKRI (LL20) and VQRIKDFLRNLVPRTES (VQ17) (table S8). Together with the finding that HtrA cleaves LL-37 near its C terminus, we concluded that HtrA cleaves LL-37 specifically between isoleucine at position 20 (Ile^20^) and valine at position 21 (Val^21^) (Fig. 4F). To further validate this specific cleavage site, we synthesized three LL-37 mutants, including LL-37^I20Q^, in which Ile^20^ was substituted with glutamine; LL-37^V21S^, in which Val^21^ was substituted with serine; and a double-point mutant LL-37^I20Q/V21S^ containing both mutations. We found that the single-point mutants LL-37^V21S^ and LL-37^I20Q^ were still cleaved by HtrA (fig. S4, B and C), whereas the double-point mutant LL-37^I20Q/V21S^ completely prevented cleavage by HtrA (Fig. 4G). Furthermore, we evaluated the antibacterial activity of two cleavage products, LL20 and VQ17, against 17 *C. jejuni* clinical isolates, and found that both peptides completely abolished their antibacterial activity (fig. S4D and table S9), indicating that HtrA protects *C. jejuni* from LL-37 killing by cleaving it into two inactive peptides.

To gain a deeper understanding of the diversity and distribution of HtrA, we performed a sequence alignment of HtrA proteins across all LL-37–sensitive and –resistant *C. jejuni* clinical isolates. Maximum likelihood phylogenetic analysis revealed that HtrA is highly conserved and widespread in *C. jejuni* (fig. S5A), implying that the ability of HtrA to cleave LL-37 appears to be common among different isolates. This notion is supported by previous transcriptomic analysis showing that *htrA* expression was up-regulated in the LL-37–sensitive 81-176 strain in response to LL-37 (table S10). To further confirm this possibility, we cloned and purified recombinant HtrA from LL-37–sensitive 81-176 strain, and then incubated it with LL-37. As expected, HtrA cloned from 81-176 strain was indeed capable of cleaving LL-37 (fig. S5B). This raised a question of why LL-37–sensitive isolates fail to effectively counteract the antimicrobial activity of LL-37 despite harboring an HtrA capable of cleaving it. To address this question, we randomly selected four LL-37–sensitive and –resistant isolates and quantified the levels of HtrA secretion in these isolates under LL-37 treatment by WB. As shown in fig. S5C, HtrA was barely detectable in LL-37–sensitive isolates, whereas it was highly secreted in LL-37–resistant isolates. These findings suggest that the resistance of *C. jejuni* to LL-37 is closely associated with the level of HtrA secretion, highlighting the pivotal role of HtrA in protecting the bacteria from LL-37 killing and facilitating intestinal colonization.

### NssR positively regulates *htrA* expression by binding to P*htrA* promoter

**Fig. 5.**
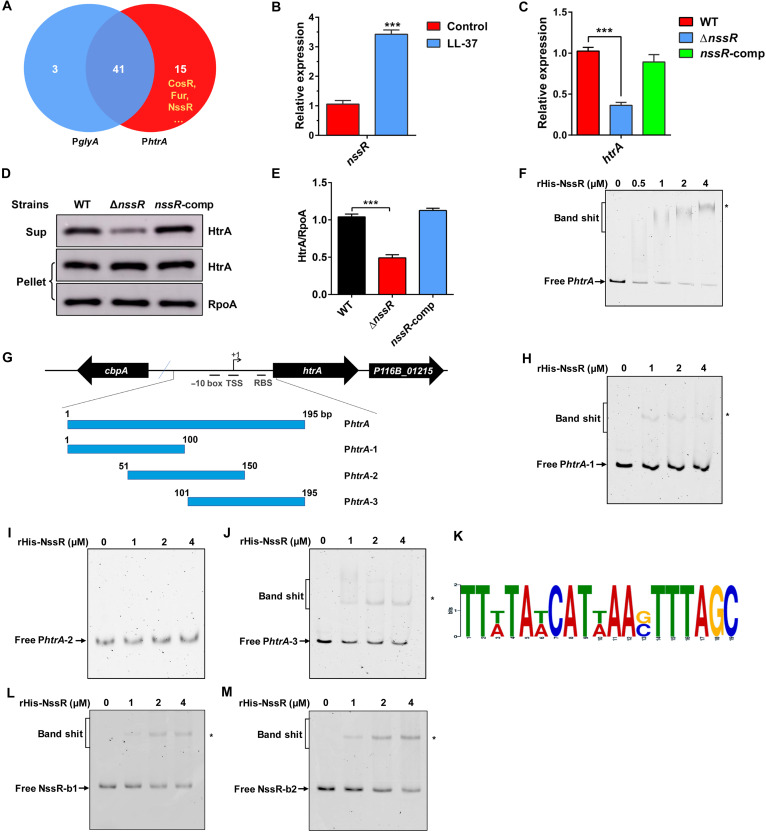
NssR confers *C. jejuni* resistance to LL-37 by directly up-regulating *htrA* expression. (**A**) Venn diagram of the number of proteins identified in DNA pull-down assay. Only 15 proteins appear to interact with P*htrA*. (**B**) qRT-PCR analysis of *nssR* gene in P116B strain exposed to LL-37 (11 μg/ml). Error bars represented the SD of independent replicates. Data were analyzed using Student’s *t* test (***P < 0.001). (**C**) qRT-PCR analysis of *htrA* gene in WT, *nssR* deletion, and complementary strains in response to LL-37 (5.5 μg/ml). Error bars represented the SD of independent replicates. Data were analyzed using Student’s *t* test (***P < 0.001). (**D** and **E**) WB (D) and densitometry analysis (E) of HtrA in P116B strain supernatants and pellets in the presence of LL-37 (5.5 μg/ml). RpoA was used as a loading control. Error bars represented the SD of independent replicates. Data were analyzed using Student’s *t* test (***P < 0.001). (**F**) EMSAs of NssR with the P*htrA* promoter. The fluorescently labeled P*htrA* promoter was incubated with increasing concentrations of rHis-NssR. DNA-protein complexes are indicated by an asterisk. (**G**) A diagram of the P*htrA* promoter and the three regions carried on the DNA fragments used in this assay. Fragment names are displayed in the figure. (**H** to **J**) EMSA analysis of NssR-specific binding sites on the P*htrA* promoter. All three FAM-labeled fragments (4 nM) (G) were incubated with purified rHis_6_-NssR. DNA-protein complexes are indicated by an asterisk. (**K**) Conserved motifs of NssR on P*htrA*. A MEME analysis of the two sequence segments highlighted within the blue boxes in (fig. S6E). (**L** and **M**) EMSA analysis of NssR binding to the NssR-b1 (L) and NssR-b2 (M). Two FAM-labeled fragments (4 nM) were incubated with purified rHis_6_-NssR. DNA-protein complexes are indicated by an asterisk.

Given that the expression level of HtrA is critical for *C. jejuni* to resist LL-37 killing, we next sought to explore the regulatory mechanisms controlling *htrA* expression. To this end, we carried out a DNA pull-down assay using the P*htrA* promoter as a bait to capture potential direct transcriptional regulators from cellular lysates of P116B strain. MS analysis showed that 15 proteins had potential specific interactions with the P*htrA* promoter (Fig. 5A). Among these, four proteins (CosR, NssR, Fur, and Cj1000) were predicted to be transcription factors with DNA binding activity (table S11). Intriguingly, our transcriptomic analysis revealed that *nssR* (*P116B_00456*) expression was also significantly up-regulated in response to LL-37, which was further confirmed by qRT-PCR (Fig. 5B and table S5). These results indicate that NssR may function as a direct regulator of *htrA*, thereby mediating the bacterial response to LL-37.

To examine whether NssR has a role in regulating *htrA* expression, we generated *nssR* deletion and complementation strains. Using qRT-PCR, we found that deletion of *nssR* resulted in a significant reduction in *htrA* expression (~2.8-fold), while complementation of a functional *nssR* to the *nssR* mutant restored the expression of *htrA* to WT levels (Fig. 5C). We next performed WB analyses to determine whether NssR promotes the extracellular secretion of HtrA and found that HtrA secretion was reduced by approximately 2.1-fold in the Δ*nssR* mutant in response to LL-37 (Fig. 5, D and E). These findings suggest that NssR functions as a positive regulator of *htrA*. Furthermore, we determined the MICs of LL-37 against both strains. In consistent with the reduced expression of *htrA*, Δ*nssR* mutant exhibited a markedly lower MIC relative to WT (table S7), which is unlikely to result from growth defects, as deletion of *nssR* did not affect bacterial growth (fig. S6A). To determine whether NssR directly regulates *htrA* expression, we analyzed the interaction of NssR with P*htrA* promoter by electrophoretic mobility shift assays (EMSAs). As shown in Fig. 5F, with increasing concentrations of NssR protein, slowly migrating bands were observed for the P*htrA* promoter. The specific binding of NssR to P*htrA* promoter was further confirmed by competitive EMSAs (fig. S6B), indicating that NssR directly up-regulates *htrA* expression by interacting with the P*htrA* promoter. To further identify the specific binding sites of NssR in the P*htrA* promoter, we divided the P*htrA* promoter into three overlapping fragments with 50–base pair overlaps between adjacent segments (Fig. 5G). The binding between the DNA fragments and purified NssR protein was analyzed using EMSAs. We observed that NssR binds to P*htrA*-1 and P*htrA*-3, but not to P*htrA*-2, suggesting that NssR has two distinct NssR-binding sites within the P*htrA* promoter, which are located in the P*htrA*-102/−51 and P*htrA*-1/+93 sequences, respectively (Fig. 5, H to J). The specificity of NssR binding to P*htrA*-1 and P*htrA*-3 was further confirmed by competitive EMSA assays (fig. S6, C and D). We next applied MEME to both sequences and identified a conserved NssR-binding motif: TTWTAWCATWAASTTTAGC (Fig. 5K and fig. S6E). To test whether NssR directly binds to the two predicted binding sites, we synthesized FAM-labeled DNA fragments corresponding to the conserved motif regions, designated NssR-b1 and NssR-b2, and performed EMSA assays. NssR bound specifically to both sites in a concentration-dependent manner (Fig. 5, L and M), and the binding specificity was further confirmed by competitive EMSA (fig. S6, F and G), indicating that NssR mediates activation of *htrA* by acting on this conserved motif. Together, these findings support the conclusion that NssR confers resistance to LL-37 in *C. jejuni* by directly up-regulating *htrA* expression.

### Noncleavable LL-37^I20M/V21R^ mutant effectively reduces *C. jejuni* intestinal colonization

**Fig. 6.**
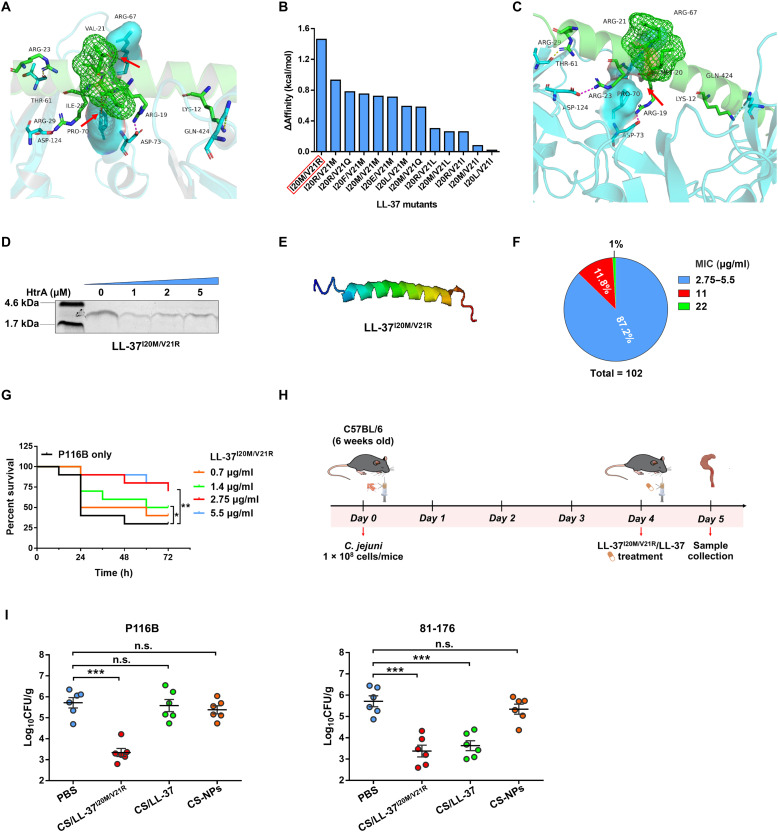
Noncleavable LL-37^I20M/V21R^ mutant effectively reduces *C. jejuni* intestinal colonization. (**A**) Docking result of HtrA with LL-37 by molecular docking simulations. The van der Waals interactions, highlighted by the red arrows, are represented by red dashed lines. (**B**) The top 13 ΔAffinity results of the LL-37 double point mutants with HtrA. (**C**) Docking result of HtrA with LL-37^I20M/V21R^ by molecular docking simulations. The van der Waals interactions highlighted by the red arrow disappeared. (**D**) SDS-PAGE analysis of LL-37^I20M/V21R^ cleavage after incubation with HtrA for 2 hours. (**E**) 3D modeling of LL-37 ^I20M/V21R^ using I-TASSER. (**F**) Pie chart indicating the MIC results of LL-37^I20M/V21R^ against 102 clinical isolates of *C. jejuni*. Blue, red, and green indicate MIC values of 2.75 to 5.5, 11, and 22 μg/ml, respectively. (**G**) Survival curves of *G. mellonella* larvae infected with *C. jejuni* P116B strain and treated with LL-37 ^I20M/V21R^ over 72 hours. A statistical analysis of survival curves was performed using the log-rank test. Significant differences in survival are indicated as *P < 0.05 or **P < 0.01. (**H**) Schematic of the in vivo experiments. C57BL/6 mice were orally infected with *C. jejuni* (10^8^ bacterial cells per mouse) on day 0. Four days postinfection, the mice were treated with PBS, CS/LL-37^I20M/V21R^-NPs, or CS-NPs. The mice were euthanized on day 5, and cecum contents were collected bacterial enumeration. Syringe illustration credit: G. D’ottavio, Scidraw.io. Created in BioRender. Li, X. (2026) https://BioRender.com/r49s6mw. (**I**) Bacterial counts recovered from cecum contents of the orally *C. jejuni* infected mice treated with PBS (Control), CS/LL-37^I20M/V21R^-NPs (2.75 μg/ml for P116B and 81-176), CS/LL-37-NPs (88 μg/ml for P116B, or 11 μg/ml for 81-176), or CS-NPs. Data are means ± SEM from three independent repeats (*n* = 6 animals per group). Bars represent mean CFU of all mice. Statistical significance was analyzed using two-way ANOVA followed by multiple comparisons: ***P < 0.001; n.s., not significant.

Having established that HtrA-mediated cleavage of LL-37 contributes to *C. jejuni* intestinal colonization, we next wondered whether a noncleavable LL-37 analog could more effectively combat *C. jejuni* infection, particularly LL-37–resistant isolates. Strikingly, the LL-37^I20Q/V21S^ mutant, which completely prevented HtrA cleavage (Fig. 4G), did not exhibit enhanced antibacterial efficacy in vitro compared to the WT LL-37 (table S12). To rationally design an LL-37 analog that can overcome HtrA cleavage and exhibit improved antibacterial activity, we performed molecular docking simulations to analyze the specific interaction between HtrA and LL-37. The HtrA structure was predicted using AlphaFold2, and the monomeric structure of LL-37 was retrieved from the Protein Data Bank (PDB: 5NMN) (fig. S7, A and B). The most stable conformation identified in the docking simulations was selected for further analysis. (table S13). Notably, two distinct van der Waals interactions were identified in the docking model: one between Ile^20^ of LL-37 and Pro^70^ of HtrA, and another between Val^21^ of LL-37 and Arg^67^ of HtrA (Fig. 6A). This further confirms that HtrA directly recognizes and interacts with the Ile^20^ and Val^21^ sites of LL-37, which were identified as cleavage sites in our in vitro assays (Fig. 4F).

To develop an LL-37 analog with reduced binding affinity to HtrA, we performed saturation mutagenesis at the Ile^20^ and Val^21^ sites of LL-37, generating a total of 482 analogs. Among them, 22 analogs were predicted to exhibit improved structural stability compared to the WT LL-37 (ΔStability <0) (table S14). LL-37^I20Q/V21S^ mutant was not included in these analogs, which could account for its limited antibacterial activity despite its ability to prevent HtrA cleavage (Fig. 4G). Subsequently, each of these stable analogs was subjected to molecular docking simulations with HtrA to evaluate the impact of residue variation on binding affinity. We found that LL-37^I20M/V21R^ mutant displayed the lowest binding affinity to HtrA, with a reduction of 1.46 kcal/mol in binding energy (Fig. 6B and table S14). Molecular docking simulations further revealed that this mutant disrupted the van der Waals interactions observed in the WT LL-37-HtrA complex (Fig. 6C). To validate these predictions, we performed cleavage assays and confirmed that the LL-37^I20M/V21R^ mutant completely blocked HtrA-mediated cleavage (Fig. 6D). We further characterized the biochemical properties of LL-37^I20M/V21R^ mutant, revealing that it retained α helix structure of WT LL-37 (Fig. 6E), while exhibiting an increased net charge of +7, a higher molecular weight (4.57 kDa), and an elevated isoelectric point (pI) (table S15). Collectively, these results indicate that the LL-37^I20M/V21R^ mutant combines structural stability with reduced HtrA binding, rendering it a promising candidate for enhanced antibacterial activity against *C. jejuni*.

LL-37^I20M/V21R^ mutant showed a 32-fold decrease in MIC against the LL-37–resistant P116B strain compared to WT LL-37 (table S1). In addition, MIC assays performed on the remaining 101 *C. jejuni* clinical isolates revealed that LL-37^I20M/V21R^ mutant exhibited significantly enhanced antibacterial activity, with MIC values ranging from 2.75 to 22 μg/ml, and 99% (101 of 102) of the tested strains showed MIC values of ≤11 μg/ml (Fig. 6F and table S1), strongly confirming its enhanced antibacterial efficacy across a broad spectrum of *C. jejuni* isolates in vitro. The antibacterial efficacy of LL-37^I20M/V21R^ mutant was further validated in vivo using a *G. mellonella* infection model. The results showed that LL-37^I20M/V21R^ mutant increased the survival rate from 30 to 60% at a concentration of 2.75 μg/ml (Fig. 6G and fig. S7C), which stands in stark contrast to our previous findings that WT LL-37 failed to significantly enhance larval survival even at high concentrations of 88 μg/ml (Fig. 3A). To further assess whether LL-37^I20M/V21R^ mutant could reduce the colonization of *C. jejuni* in the intestine, we prepared an LL-37^I20M/V21R^–loaded chitosan nanoparticles (CS/LL-37^I20M/V21R^-NPs) that exhibited comparable biochemical activity to the CS/LL-37-NPs (fig. S8) and then evaluated their efficacy in a mouse infection model (Fig. 6H). Our results revealed that CS/LL-37^I20M/V21R^-NPs significantly reduced colonization of the LL-37–resistant P116B strain in the cecum by 2.372 log_10_ CFU/g compared to the phosphate-buffered saline (PBS) group (*P* < 0.001) (Fig. 6I). A similar reduction of 2.337 log_10_ CFU/g was observed for LL-37–sensitive 81-176 strain (Fig. 6I). Together, these findings demonstrate that noncleavable LL-37^I20M/V21R^ mutant can efficiently facilitate intestinal decolonization of *C. jejuni*, underscoring its potential as a promising therapeutic agent for the treatment of *C. jejuni* infection.

## DISCUSSION

**Fig. 7.**
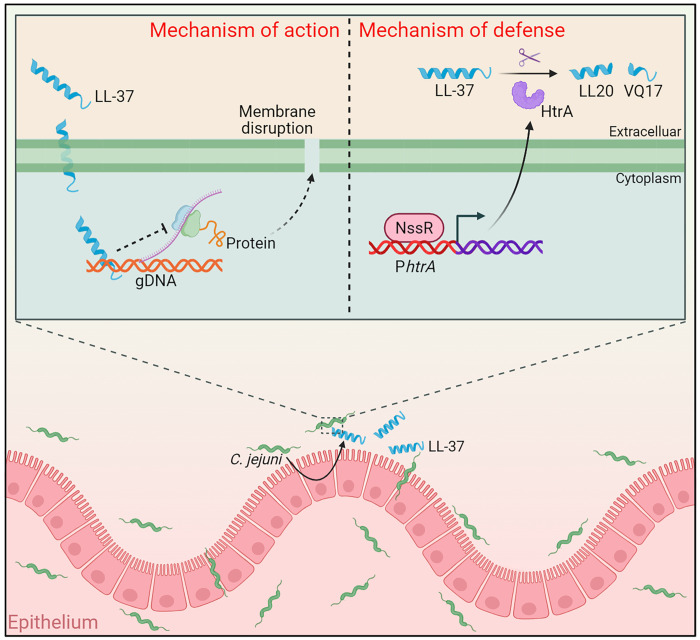
Model of the interaction between *C. jejuni* and LL-37. Upon *C. jejuni* infection, intestinal epithelial cells secrete LL-37 as part of the host defense response. Two possible outcomes could occur under this scenario. One, LL-37 penetrates the bacterial membrane and subsequently binds to the genomic DNA of *C. jejuni*, thereby disrupting essential biological processes and ultimately leading to cell death. Two, to evade this host defense, *C. jejuni* activates the NssR regulatory pathway, which directly up-regulates *htrA* expression. The secreted HtrA protease specifically cleaves LL-37 into two inactive fragments, abolishing its antimicrobial activity and thus promoting successful *C. jejuni* colonization of the intestinal epithelium. Created in BioRender. Li, X. (2026) https://BioRender.com/z8mx573.

The human cathelicidin LL-37 is a crucial component of the innate immune system defending bacterial infections (*33*). However, how *C. jejuni*, a major gastrointestinal pathogen, responds to LL-37 during host colonization remains unclear. In this study, we characterized and proposed a model detailing the mechanism by which LL-37 interacts with *C. jejuni* (Fig. 7). Specifically, upon initial intestinal colonization, the host epithelium induces the expression of LL-37 to eliminate invading *C. jejuni* by interfering with essential intracellular processes and disrupting membrane integrity. *C. jejuni* has in turn evolved a unique and efficient strategy to evade LL-37 killing. The transcriptional regulator NssR is activated by LL-37 and directly promotes *C. jejuni* to secrete a serine protease HtrA, which degrades LL-37 into two inactive fragments, and consequently defending against LL-37 killing to facilitate the intracellular survival of *C. jejuni*. On the basis of these findings, we designed an LL-37 analog, LL-37^I20M/V21R^, which cannot be cleaved by HtrA, and demonstrated its robust antibacterial activity against *C. jejuni* both in vivo and in vitro.

It has been shown that the expression of LL-37 in most epithelial cells is constitutive and can be induced to a high concentration in response to bacterial infections (*34*–*36*). Consistent with these findings, we detected a high level of LL-37 expression in human colon epithelial Caco-2 cells after being infected with *C. jejuni*, supporting its crucial role in contributing to host innate defense. We demonstrated that LL-37 exhibits potent bacteriostatic activity against most of *C. jejuni* clinical isolates both in vivo and in vitro. Several studies have also shown its broad-spectrum activity against numerous Gram-negative and Gram-positive bacteria, with MIC ranges reported for *E. coli* (9 to 128 μg/ml), *Staphylococcus aureus* (14 to 256 μg/ml), and *Salmonella* spp. (4 to 143.7 μg/ml) (*37*–*39*). Here, we observed low MIC values in the range of 5.5 ≥ 88 μg/ml for LL-37 against *C. jejuni* clinical isolates, further underscoring its broad-spectrum inhibitory potential and promising preventive strategy. The mechanism of action of LL-37 varies among bacteria, with the primary mechanism being membrane disruption. Upon binding to the microbial membrane, LL-37 inserts into the lipid bilayer and forms transmembrane pores, thereby compromising membrane integrity and ultimately leading to cell lysis and death (*40*–*42*). By contrast, our super-resolution microscopy and RNA-seq results reveal that LL-37 rapidly penetrates membrane of *C. jejuni*, where it subsequently strongly interacts with bacterial DNA to inhibit replication and impede downstream transcription-translation of genes essential for membrane biogenesis, ultimately contributing to membrane disruption and cell death. A similar finding was also observed in *E. coli*, in which LL-37 was shown to disrupt DNA diffusion and inhibit ribosomal function (*16*). While many CAMPs exert their bactericidal effects through membrane disruption (*13*, *43*), our findings expand our understanding of the molecular targets of LL-37, suggesting that intracellular inhibitory activity may represent a common mechanism underlying its bactericidal activity.

Although LL-37 has potent and broad-spectrum antibacterial activity against various pathogens, many bacteria have acquired diverse strategies to resist LL-37 killing to facilitate their colonization and survival (*11*, *12*, *22*), reflecting a dynamic host-pathogen evolutionary arms race. We identified the serine protease HtrA as the key determinant responsible for LL-37 resistance in *C. jejuni* through cleavage of LL-37 into two inactive peptides. Results from mouse infection model further showed that HtrA contributes to intestinal colonization of *C. jejuni* when challenged with LL-37. Previous work showed that HtrA promotes bacterial colonization and invasion through cleaving tight junction proteins such as occludin, E-cadherin, and claudin-8 (*31*, *44*, *45*), whereas our results reveal a function of HtrA in contributing to bacterial colonization by inactivating the AMP LL-37. In group A *Streptococcus* (GAS), the protease ScpC also cleaves LL-37 but generates fragments that retain bactericidal activity. Instead of neutralizing LL-37, ScpC-mediated cleavage prevents LL-37 from activating P2X7R and EGFR signaling pathways, which are essential for the host defense against GAS infection (*15*). These findings highlight that bacteria have evolved distinct proteolytic strategies as adaptive mechanisms to counteract LL-37 pressure during infection. Bioinformatics analysis further revealed that the HtrA protease is widely distributed and conserved in *C. jejuni* clinical isolates, indicating that HtrA-mediated cleavage represents a common strategy used by this species to resist LL-37 killing. This notion is further supported by the observation that HtrA proteins cloned from both LL-37–sensitive and –resistant isolates displayed comparable cleavage activity toward LL-37 in vitro. We propose that the varying susceptibilities of *C. jejuni* isolates to LL-37 likely result from the differential secretion levels of HtrA, as LL-37–resistant isolates showed a markedly higher HtrA secretion. These findings dovetail nicely with previous work showing that the HtrA secretion was low in LL-37–sensitive 81-176 strain and decreased in a time-dependent manner (*46*). In addition, HtrA-mediated cleavage of LL-37 is not restricted to *C. jejuni* and might be a broader phenomenon in different bacterial genera. For example, in *E. coli*, HtrA homologs DegP and DegQ preferentially cleave peptides at the Ile/Xaa or Val/Xaa sites (*47*), which closely resemble the Ile^20^ and Val^21^ cleavage sites on LL-37 identified for *C. jejuni* HtrA.

Increasing evidence suggests that HtrA plays an important role in *C. jejuni* adaptation to environmental stress, such as heat and oxygen stress, during intestinal colonization (*45*, *48*). Nevertheless, the regulatory mechanisms controlling its expression remain unknown. In this study, we report that NssR, a member of the Crp-Fnr superfamily transcriptional regulator (*49*), directly promotes *htrA* expression by binding to its promoter. Our results showed that NssR regulates not only *htrA* expression but also the secretion of HtrA into the extracellular environment. Considering that HtrA secretion is greater in the LL-37–resistant isolates than in the susceptible isolates, we speculate that LL-37–resistant isolates may exhibit stronger or more sustained activation of NssR upon LL-37 exposure, leading to enhanced HtrA expression and secretion. This notion is supported by our RNA-seq data, which show a higher level of *nssR* expression in LL-37–resistant isolates compared with susceptible isolates after LL-37 treatment (SRA accession numbers: PRJNA1370360 and PRJNA1371734). In addition, NssR may influence pathways associated with protein export, thereby promoting efficient secretion of HtrA. The high induction of *nssR* expression upon exposure to LL-37 implies that NssR may function as a direct sensor of LL-37. Although AMPs sensing in bacteria is typically mediated by two-component regulatory systems (TCS) (*50*–*53*), *C. jejuni* appears to lack a TCS capable of sensing LL-37 because our RNA-seq data showed that none of the known TCS (*54*) were significantly up-regulated following LL-37 treatment. Instead, given that NssR is a major regulator of *C. jejuni* behavior in response to nitrosative stress (*49*, *55*, *56*), we propose that LL-37 may induce the intracellular generation of nitric oxide or cyclic nucleotide second messengers such as cAMP, c-di-GMP, or cGMP, which serve as signaling molecules sensed by NssR to activate *htrA* expression (*57*, *58*). However, further investigations are warranted to elucidate the molecular mechanism by which NssR senses and transduces LL-37–associated signals.

Owing to the rapid and broad-spectrum bactericidal activity, AMPs are considered a promising alternative to conventional antibiotics (*59*). However, the rapid evolution of bacteria to evade host-derived AMPs underscores the need to develop optimized AMPs for effective pathogen clearance. Inspired by our findings that HtrA facilitates *C. jejuni* intestinal colonization by cleaving LL-37, we designed a noncleavable LL-37 analog, LL-37^I20M/V21R^, to overcome this bacterial defense strategy. Through combined in vitro and in vivo antimicrobial tests, we confirmed the high therapeutic efficacy of LL-37^I20M/V21R^ against *C. jejuni* infection, particularly against LL-37–resistant clinical isolates. LL-37^I20M/V21R^ also exhibited substantially enhanced antibacterial activity against other enteric pathogens, such as *E. coli*, *Salmonella*, and *S. aureus*, relative to LL-37 (table S16). Given the diverse composition of the gut microbiota, which comprises both beneficial and pathogenic bacteria (*60*), we further observed that neither LL-37^I20M/V21R^ nor native LL-37 inhibited probiotic bacteria in vitro, including *Lactobacillus* spp. and *Clostridium butyricum* (table S16), suggesting a selective antimicrobial profile that targets pathogens while sparing beneficial gut microbes. These results position LL-37^I20M/V21R^ as a promising candidate for the treatment of enteric bacterial infections, offering a crucial alternative in the context of increasing multidrug resistance (*61*). Nevertheless, further in vivo studies are required to evaluate the effects of LL-37^I20M/V21R^ on gut microbial homeostasis.

In conclusion, our work presents comprehensive analysis of the interaction patterns between *C. jejuni* and LL-37: First, in response to *C. jejuni* infection, LL-37 is induced to bind to bacterial genome DNA, effectively inhibiting essential cellular processes and hence promoting bacterial clearance; conversely, pathogenic *C. jejuni* has evolved a serine protease HtrA that directly cleaves LL-37 by recognizing Ile^20^ and Val^21^ residues, thereby facilitating bacterial colonization and survival in the intestine. These findings establish a mechanistic understanding of how AMPs act against *C. jejuni* and highlight potential avenues for the development of strategies to combat this pathogen’s infections.

## MATERIALS AND METHODS

### Bacterial strains and plasmids

All strains and plasmids used in this study are listed in table S17. In short, *C. jejuni* strains were cultured under microaerobic conditions at 42°C. *E. coli* strains grow in LB broth or LB agar at 37°C (*62*). As required, antibiotics were added to the culture medium of *C. jejuni* or *E. coli* at the following concentrations: ampicillin (100 μg/ml), kanamycin (50 μg/ml), or chloramphenicol (20 μg/ml). The plasmid pMD-19T (Simple) (TaKaRa, Dalian, China) was used as a suicide vector in cloning and strain construction. Plasmid pRK2013 [American Type Culture Collection (ATCC) 37159] is an auxiliary plasmid for triparental mating conjugation, while pUOA18 is a shuttle vector of *C. jejuni* provided by Qijing Zhang (Iowa State University, Ames, USA).

### Recombinant protein purification and peptide synthesis

All the peptides used in the study, namely LL-37, LL-37^I20Q^, LL-37^V21S^, LL-37^I20Q/V21S^, LL-37^I20M/V21R^, scrambled LL-37 (Scrm), LL20, VQ17, and CRAMP, were commercially synthesized by AnHui JYHX CO. LTD, Hefei, China, with a purity of more than 95%. FITC-labeled LL-37 was also synthesized from the same company (table S17). Recombinant HtrA was cloned, expressed, and purified using the *E. coli* strain BL21(DE3). The *htrA* or *htrA^S225A^* genes from *C. jejuni* P116B strain and the *htrA* gene from 81-176 strain were amplified and inserted into the pCold I vector at *B*amH I and *S*al I sites using the ClonExpress II one-step cloning kit (Vazyme, Nanjing, China). All clones were verified by sequencing before protein purification. The induced proteins were purified via affinity chromatography using a HiTrap Ni^2+^-chelating column, following the manufacturer’s instructions for the His Bind Purification Kit (Novagen, EMO Millipore Corp, Billerica, MA, United States). Purified proteins were stored at −70°C. Primers used for strain construction are listed in table S18.

### WB and preparation of polyclonal antiserum against HtrA

To assess the protein abundance of LL-37, a WB assay was performed with an anti–LL-37 antibody (Santa Cruz Biotechnology, China) in Caco-2 cells with or without *C. jejuni* 81-176. The anti–glyceraldehyde-3-phosphate dehydrogenase antibody (Sangon Biotech, China) was used for protein loading normalization. To analyze *htrA* secretion, polyclonal antiserum against HtrA was prepared as previously described (*63*). In brief, purified HtrA-His_6_ protein was administered intradermally multiple times to 6-week-old female BALB/c mice (VITAL RIVER, Beijing, China) in combination with an equal volume of Freund’s complete adjuvant (Sigma-Aldrich, Darmstadt, Germany) to generate a polyclonal antiserum. In addition, to strengthen the immune response, 10 μg of HtrA-His_6_ protein emulsified in Freund’s incomplete adjuvant (Sigma-Aldrich, Darmstadt, Germany) was injected every 2 weeks. The untreated group was injected with PBS instead of HtrA-His_6_ protein. Subsequently, blood samples were collected from each treated group, and serum samples were separated and stored at −70°C. The specificity of the antiserum was assessed using WB analysis.

HtrA expression was assessed as previously described (*64*). Briefly, equal amounts of supernatants and pellets from *C. jejuni* cells treated with or without LL-37 were separated by SDS–polyacrylamide gel electrophoresis (SDS-PAGE, Solarbio, China) and transferred onto nitrocellulose membranes. After blocking with 2% bovine serum albumin in tris-buffered saline containing Tween 20 (TBST) buffer for 2 hours, membranes were incubated overnight at 4°C with anti-HtrA primary antibody (1:5000). Following washing with TBST, membranes were incubated for 1 hour with a goat anti-mouse immunoglobulin G secondary antibody (1:1000; Cell Signaling Technology, USA). Results were analyzed using a gel imaging system (Bio-Rad Laboratories, USA). The anti-RpoA antibody was used for protein loading normalization.

### Evaluation of MIC and MBC

MIC and MBC of LL-37 and its derived analogs for *C. jejuni* strains were determined as described previously (*8*, *65*). Briefly, for MIC, *C. jejuni* strains were cultured overnight in Mueller-Hinton (MH) broth. The overnight cultures were diluted to an optical density at 600 nm (OD_600_) of 0.05 and inoculated into 96-well microtiter plates containing 100 μl of MH broth supplemented with selected AMP concentrations. The plates were incubated at 37°C under microaerobic conditions with constant shaking, and the absorbance at 600 nm (*A*_600_) was measured after 24 hours using a Synergy-Mx microplate reader (BioTek, USA). Each experiment was performed in triplicate, including positive controls (without AMPs) and negative controls (without bacteria). For the MBC determination, 10 μl of samples were taken from the wells that showed no growth and streaked onto *Campylobacter* blood-free selective agar containing charcoal cefoperazone deoxycholate (CCDA) (Oxoid, Basingstoke, United Kingdom). The MBC was defined as the lowest concentration of AMPs that exhibited no bacterial growth after 48 hours of incubation. To further characterize the antimicrobial spectrum of LL-37 and LL-37^I20M/V21R^, MIC assays were performed against a broader panel of bacterial species following established protocols for each organism. These included *E. coli* ATCC 25922 (*66*), *Salmonella* C50336 (*67*), and *S. aureus* USA300 (*68*). MIC assays were also conducted for probiotic strains maintained in our laboratory, including *Lactobacillus* isolates Q2 and Q3 (*69*) and *C. butyricum* isolates Y2 and Y35 (*70*), using growth media and incubation conditions optimized for each species.

### Growth curve assay

*C. jejuni* P116B, *htrA* and *nssR* mutants, and complementary strains were cultured in MH broth (BD, USA) to an OD_600_ of 0.1 at 42°C, with growth monitored by OD_600_ over 24 hours. For LL-37 activity against *C. jejuni* 81-176, cultures at OD_600_ 0.1 were treated with 1× or 2× MIC (5.5/11 μg/ml) at 37°C. Growth kinetics were assessed by OD_600_ and bacterial counts (log_10_ CFU/ml) at specified intervals over 12 hours. CS/LL-37-NPs (5.5 μg/ml for 81-176; 88 μg/ml for P116B), CS/LL-37^I20M/V21R^-NPs (2.75 μg/ml for P116B), or blank CS-NPs control were tested similarly. Results are reported as means ± SEM from three individual tests. Data were analyzed using Student’s *t* test (*P < 0.05, **P < 0.01, ***P < 0.001).

### RNA extraction and quantitative real-time PCR

*C. jejuni* strains 81-176 and P116B strains were cultured in MH broth to an OD_600_ of 0.1 and grown at 42°C with shaking (120 rpm) to logarithmic phase (OD_600_ ≈ 0.5). Cultures were then diluted to OD_600_ 0.1 and incubated with LL-37 (81-176: 5.5 μg/ml; P116B: 11 μg/ml) or left untreated for 2 hours at 37°C with shaking. Total RNA was extracted using the RNeasy Plus Mini Kit (Qiagen, Germany), and cDNA was synthesized with the RT Reagent Kit (TaKaRa, China). qRT-PCR was performed using FastStart Universal SYBR Green Master (ROX) (Roche, Germany) on an ABI PRISM 7500 system (Applied Biosystems, USA) (*64*). The *glyA* gene was used as an endogenous control. The results are expressed as means ± SD. All primers for qRT-PCR were listed in table S18.

### RNA sequencing

RNA-Seq libraries were prepared from six bacterial samples: three biological replicates each of *C. jejuni* 81-176 and P116B, with or without LL-37 treatment. RNA quality was assessed using with a 2100 Bioanalyzer (Agilent) and an ND-2000 spectrophotometer (NanoDrop Technologies), and only high-quality samples (OD_260/280_ = 1.8 to 2.2, OD_260/230_ ≥ 2.0, and RIN ≥ 6.5) were selected for sequencing library construction (*71*). cDNA was synthesized using the SuperScript double-strand cDNA synthesis kit (Invitrogen, USA). Library quality was verified by Agilent 2100 Bioanalyzer, and sequencing was performed on an Illumina HiSeq 4000 platform. Sequencing was biologically replicated in a separate experiment conducted by Majorbio (Shanghai, China). Genes with *P* < 0.05 were considered significant, and data were analyzed on the Majorbio cloud platform (https://cloud.majorbio.com). COG and KEGG analyses grouped these significantly expressed genes into various functional groups (*72*).

### Scanning electron microscopy

The ultrastructural effects of LL-37 on *C. jejuni* cells were examined using SEM. The *C. jejuni* 81-176 cells were treated with LL-37 at a concentration of 2 × MIC for 15 min at 37°C. Following treatment, the cells were centrifuged at 4000*g* for 10 min at 4°C and washed three times with PBS buffer. The treated and control *C. jejuni* cells were then fixed in 2.5% glutaraldehyde at 4°C for 12 hours. After fixation, the samples underwent a gradient dehydration process in ethanol solutions of 30, 50, 70, 80, 90, and 100%, with each step lasting 15 min. Last, the cells were dried and coated with gold before imaging with an SEM (S-4800, Hitachi, Tokyo, Japan) (*73*). The CS/LL37^I20M/V21R^-NPs or CS/LL37-NPs were freeze-dried before imaging with an SEM (S-4800, Hitachi, Tokyo, Japan).

### Leakage of nucleic acids and proteins

The impact of LL-37 on the permeability of the *C. jejuni* cell membrane was evaluated by measuring the release of intracellular nucleic acids and proteins. Suspensions of *C. jejuni* strain 81-176 were prepared at a concentration of 10^7^ CFU/ml and treated with LL-37 at 2 × MIC. The cells were then incubated at 37°C for 2, 3, 5, and 7 hours. The leakage of nucleic acids and proteins was assessed by measuring *A*_260_ and *A*_280_, respectively.

### MTT assay

The 3-(4,5-dimethylthiazol-2-yl)-2,5-diphenyltetrazolium bromide (MTT) assay was conducted to determine cytotoxicity in vitro (*74*). Cells were seeded into 96-well plates at a density of 1 × 10^5^ cells per well and incubated for 24 hours. Subsequently, 100 μl of LL-37 or LL-37^I20M/V21R^ at various concentrations was added to each well, with 1% dimethyl sulfoxide as the control. After incubation at 37°C for 2 hours, MTT reagent was added following the protocol MTT of Cytotoxicity Assay Kit (Solarbio, China). The wells were then incubated for an additional 4 hours, washed once with PBS, and treated with formazan solvent. Once the formazan crystals were fully dissolved, the solution was diluted twofold, and *A*_570_ was measured using a microplate reader to quantify cell viability. Data were recorded and analyzed accordingly.

### Confocal laser-scanning microscopy

The *C. jejuni* cells in the mid-logarithmic phase were treated with FITC-labeled LL-37 (MIC) at 37°C for 30 min and then centrifuged at 4000*g* for 10 min at 4°C. The samples were then centrifuged at 4000*g* for 10 min at 4°C and washed three times with PBS buffer. To assess membrane permeability, the cells were resuspended in 200 μl of PBS, followed by the addition of SYTOX Orange Dead Cell Stain (S11368, Thermo Fisher Scientific) and incubation for 10 min at 4°C. Afterward, the samples were centrifuged again at 4000*g* for 10 min at 4°C and washed three times with PBS. The *C. jejuni* cells were fixed with glutaraldehyde and paraformaldehyde for 30 min at 4°C. The fluorescence was observed using a Leica TCS SP8 STED confocal fluorescence microscope (Leica Microsystems, Wetzlar, Germany).

### Super-resolution microscopy

To observe the interaction of LL-37 with *C. jejuni* strain 81-176 under a super-resolution microscope, *C. jejuni* cells were cultured to the logarithmic growth phase and then immobilized on glass coverslips treated with 0.01% poly-l-lysine (molecular weight >150,000 Da) and allowed to adsorb for 30 min. FITC-labeled LL-37 (MIC) or Sytox Orange (S11368, Thermo Fisher Scientific) was then added and set in place to begin observational imaging, recording the time point *t* = 0 at the moment of imaging. Single Slice-SIM images of *C. jejuni* cells were acquired on a Multi-SIM (Multimodality Structured Illumination Microscopy) imaging system (NanoInsights-Tech Co. Ltd.) equipped with Objective Plan-Apochromat 63×/1.40 Oil M27 (ZEISS), semiconductor lasers (405 nm)/solid-state lasers (488, 561 nm), and a complementary metal-oxide semiconductor) camera (Photometrics Kinetix). To obtain optimal images, immersion oils with refractive indices of 1.518 were used for *C. jejuni* 81-176 cells on glass coverslips. The microscope is routinely calibrated with 100-nm fluorescent spheres to calculate both the lateral and axial limits of image resolution. SIM image stacks were reconstructed using SI-Recon 2.23.3 (NanoInsights) with the following settings: pixel size, 62.6 nm; channel-specific optical transfer functions; Wiener filter constant 0.01 for 2D mode; discard Negative Intensities background. Pixel registration was corrected to be less than 1 pixel for all channels using 100-nm fluorescence beads. Time-lapse movies were captured with a duration of 1.97 min, acquiring images at a rate of one frame per second with an exposure time of 30 ms. Fluorescence intensity was quantified as the mean fluorescence of the foreground, calculated by dividing the total fluorescence signal by the foreground area identified via Otsu thresholding (*75*). The defocused fluorescence signal anomalies (*t* = 1.50 to 1.65 min) were corrected by fitting a power function *Y* = *X^a^* to the valid data using least-squares regression, where *X* represents time and *Y* represents the fluorescence intensity. After determining the fitting parameters, fluorescence values for the anomalous time points were inferred by substituting their time values into the fitted equation, ensuring accurate signal reconstruction and continuity.

### Gel retardation assay

Genomic DNA from strain 81-176 or *E. coli* ATCC25922 was extracted using the TIANamp Bacteria DNA Kit (TIANGEN, China) according to the provided instructions. To investigate the binding of LL-37 to DNA, gel retardation assays were performed. A total of 90 ng of DNA [in 10 mM tris, 1 mM EDTA buffer (pH 8.0)] was incubated with varying concentrations of LL-37 at 37°C for 1 hour. After adding the DNA loading buffer, DNA migration was evaluated through electrophoresis on a 2% agarose gel.

### HtrA cleavage assay

The cleavage of LL-37 and its derived analogs by HtrA was assessed by incubating HtrA protein with the peptides for 2 hours at 37°C. HtrA^S225A^ protein and CRAMP served as negative control. The cleavage reaction was terminated by adding 2 × tricine SDS sample buffer and heating the mixture at 90°C for 7 min. Subsequently, the samples were analyzed on 18.5% tris-tricine gels (P1320, Solarbio, China). The detection of FITC-labeled LL-37 was performed using the Gel Doc system (Bio-Rad), whereas unlabeled peptides were visualized through Coomassie blue staining (G4540, Solarbio, China) after being fixed for 30 min in a solution of 40% methanol and 10% acetic acid. The gels were then destained with distilled water until the bands became visible (*15*).

### Surface plasmon resonance

The binding affinity of HtrA protein for LL-37 was assessed using SPR analysis as described previously (*76*). The CM5 sensor chip (Cytiva) was coated with HtrA (0.2 mg/ml) in sodium acetate (10 mM, pH 4.0) at a flow rate of 10 μl/min via standard amine coupling at 25°C. SPR analysis was conducted using a Biacore X100 instrument (Cytiva). LL-37 peptide concentrations from 0 to 4 μM were subsequently flowed over the chip surface in running buffer [10 mM Hepes, 3 mM EDTA, 150 mM NaCl, and 0.05% v/v Surfactant P20 (pH 7.4)] at a rate of 10 μl/min. The chip was subsequently regenerated by injecting a regeneration buffer [glycine-HCl (pH 2.5)]. Each cycle involved a 180-s association phase, followed by a 300-s dissociation phase. The binding curves were best fitted using a 1:1 binding model to calculate the *K*_d_ values.

### Construction of *C. jejuni* mutant and complementary strains

The *htrA* or *nssR* mutant and complementary strains were constructed as previously described (*64*). To inactivate the *htrA* or *nssR* genes, we amplified their flanking regions along with the Kan^R^ cassette from the *C. jejuni* P116B genome and the plasmid pRY107. These fragments were ligated into the pMD-19T (simple) vector using a ClonExpress one step Cloning Kit (Vazyme) to construct the suicide vectors. Competent P116B cells were prepared, and the suicide plasmids were electroporated into these cells. Positive colonies were then selected on CCDA agar supplemented with kanamycin (50 mg/ml). To construct *htrA* or *nssR* complementary strain, the target genes were amplified and ligated downstream of the P*metK* promoter in a shuttle vector pUOA18. The recombinant plasmids were introduced into the *htrA* or *nssR* mutant strain via triparental mating, using *E. coli* DH5α with the pUOA18-P*metK*-*htrA* or pUOA18-P*metK*-*nssR* plasmid as the donor and DH5α (pRK2013) as the helper strain. The cells were combined in a 1:1:10 ratio (donor/helper/recipient) and then spotted onto MH agar plates and incubated overnight at 42°C under microaerophilic conditions. After incubation, the mating spots were resuspended in MH broth and plated on CCDA plates containing antibiotics. *C. jejuni* colonies were examined and verified by PCR after 3 to 5 days. The primers used for strain construction are listed in table S18.

### DNA pull-down

For the DNA pull-down assay, *C. jejuni* P116B cultures (OD_600_ adjusted to 0.1) were grown under microaerobic conditions to the logarithmic phase. After harvesting by centrifugation (5000*g*, 8 min), the cells were washed with ultrapure water, and pellets were either used immediately or stored at −80°C. Samples underwent triple freeze-thaw cycles (−80°C for 1 hour, followed by thawing on ice), and cell lysis was performed in BS/THES buffer using sonication (40% amplitude, 30s on/off, four cycles). The lysate was centrifuged to obtain the supernatant for the pull-down assay. Beads were prepared by washing with 2× B/W buffer and incubated with Biotin-P*htrA* DNA (200 to 400 ng/μl) at room temperature for 1 hour, followed by washing with TE and BS/THES buffers. Lysates were incubated with the DNA-bound beads for 30 min, followed by centrifugation at 5000*g* to remove unbound components. The bead-bound proteins were then washed and eluted with increasing concentrations of NaCl (100 mM to 1 M), and eluates were kept on ice until SDS-PAGE and silver staining were performed for further analysis. Biotin-P*glyA* was used as a negative control*.* The eluent with a high abundance of protein bands was sent to Wuhan JKR Biological Engineering Co.

### Electrophoretic mobility shift assay

5′-FAM–labeled promoter fragments were purified using the MiniBEST Agarose Gel DNA Extraction Kit (Takara, Japan). Each fragment (4 nM) was incubated with varying concentrations of NssR-His_6_ in a 20-μl buffer containing 50 mM KCl, 10 mM tris-HCl (pH 8.0), 1 mM dithiothreitol, 0.5 mM EDTA, and 5% glycerol, for 30 min at 25°C. Each reaction was verified to be specific by adding a 10-fold nonspecific competitor [Poly(dI: dC)]. For competition assays, either 20 nM (5×), 200 nM (50×), or 300 nM (75×) of unlabeled target DNA was preincubated with NssR-His_6_ for 20 min before adding 5′-FAM–labeled DNA for a 10-min incubation. Electrophoresis was conducted on a 6% nondenaturing polyacrylamide gel in 0.5× tris-borate-EDTA buffer.

### MS analyses

To identify the specific cleavage sites of HtrA on LL-37, we used HPLC-MS. A reaction mixture containing 5 μg of LL-37 was incubated with HtrA for 1 hour. The sample was centrifuged at 12,000 rpm for 20 min using a 10-kDa ultrafiltration tube, and the filtrate was collected, dried, and redissolved in 20 to 30 μl of 0.1% formic acid. The solution was desalted using a micro-desalting column, dried again, and resuspended in 10 μl of loading buffer (2% acetonitrile, 0.1% formic acid, and 98% water). After vortexing and centrifugation, the supernatant was transferred to a vial for HPLC-MS/MS analysis.

Chromatographic separation was performed on a C18 reversed-phase column (75 μm by 20 cm, 3-μm particle size), with mobile phase A consisting of 0.1% formic acid in water and mobile phase B of 80% acetonitrile with 0.1% formic acid. A gradient elution was applied, from 2 to 6% B over 3 min, followed by 6 to 20% B over 42 min, and then increasing to 32% B by 47 min and 100% B by 48 min, holding at 100% B until 60 min. The flow rate was 300 nl/min. Mass spectra were acquired in positive ion mode (ESI+) with a full-scan range of *m*/*z* 350 to 1800 using an Orbitrap mass spectrometer at a resolution of 70,000. The 20 most intense ions (charge state ≥ +2) were selected for fragmentation via high-energy collision dissociation with a normalized collision energy of 28, and fragment ions were analyzed at a resolution of 17,500.

Proteins were isolated using a pull-down assay, washed with BS/THES buffer containing Poly dI-dC (10 μg/ml), and eluted with NaCl buffers of increasing concentrations (0.1 to 1.0 M). Eluates were analyzed by SDS-PAGE and visualized by silver staining. Prominent bands were excised and sent for further analysis using a TripleTOF 5600 mass spectrometer. Data analysis was performed with IDfocus, and proteins with an unused peptide score > 1.3 (95% confidence) and at least one unique peptide were considered valid.

### Molecular dynamics simulations

Because no crystal structure of the periplasmic serine endoprotease HtrA protein has been reported, its structure in this study was predicted using Alphafold2 (*77*). The structure of the antibacterial peptide LL-37 was sourced from the PDB database under accession number 5NMN (*78*). To prepare the HtrA protein for molecular docking, it was protonated under neutral conditions (pH 7) using the H++3 server (*79*), and heteroatoms and water molecules were removed from the structure using UCSF Chimera (*80*), leaving the complete protein for further analysis. Amber14SB force field charges were assigned to the protein. Molecular docking was conducted using the HDOCK platform (*81*), which specializes in protein-protein interactions. Docking scores were evaluated with the ITScorePP empirical scoring function, where a more negative score indicates stronger binding affinity. A maximum of 100 docking conformations were generated, and the top 10 were selected for further analysis based on confidence scores. Scores greater than 0.7 were considered reliable, indicating a high likelihood of interaction. The conformation with the best docking and confidence scores was selected for subsequent analysis. PyMOL 2.04 (*82*) was used for three-dimensional (3D) structural visualization, and the academic version of Maestro (*83*) facilitated 2D interaction analysis, identifying interaction types, distances, and frequencies.

For the design and optimization of uric acid oxidase, the Rosetta 3.13 suite (*84*) was used, using an iterative mutation protocol based on side-chain conformational sampling with progressive van der Waals repulsion adjustments. Key residues were subjected to mutational analysis, where each residue was substituted with the 19 naturally occurring amino acids. A reconciling coordinate constraint was applied to keep backbone-heavy atoms near their initial positions, with the constraint gradually reduced through simulated annealing. Each mutational cycle was performed three times, and energy changes associated with each mutation were calculated. The changes in binding affinity (ΔAffinity) and protein stability (ΔStability) were then assessed to evaluate the effects of the mutations.

### Bioinformatics analysis of antibacterial peptides

The molecular weight, net charge, pI, and grand average of hydropathy (GRAVY) of LL-37 and LL-37^I20M/V21R^ were predicted using the online tool Expasy (https://web.expasy.org/). The GRAVY score represents the average hydropathy value of amino acid residues, calculated by dividing the sum of hydropathy values for all residues by the total number of residues in the protein sequence. The toxicity of the peptides was assessed using ToxinPred (http://crdd.osdd.net/raghava/toxinpred/). In addition, the tertiary structure of LL-37^I20M/V21R^ was predicted via the I-TASSER online platform (https://zhanggroup.org/I-TASSER/), and the model with the highest confidence score (C-score) was selected as the predicted structure (*85*).

### *G. mellonella* infection and survival assays

The in vivo efficacy of LL-37 or LL-37^I20M/V21R^ against *C. jejuni* was evaluated using *G. mellonella* larvae. The 81-176 or P116B strain was adjusted to an OD_600_ of 0.1 for the assays. Five groups of 10 *G. mellonella* larvae were injected with 10 μl of *C. jejuni* suspension into the last left proleg, totaling 50 larvae. After a 30-min incubation, the larvae were treated with 10 μl of LL-37 or LL-37^I20M/V21R^ at various concentrations in the last right proleg. A control group consisting of 10 larvae received 10 μl of PBS. The larvae were then incubated in the dark at 37°C in petri dishes for 72 hours, with survival assessed daily. All experiments were performed in triplicate, and the results are presented as the mean.

### Phylogenetic analysis of HtrA

On the basis of the HtrA amino acid sequences, a phylogenetic tree of HtrA proteins from 102 *C. jejuni* clinical isolates was constructed with MEGA 7.0 using the maximum likelihood method. Bootstrap values in percentages are 1000 replicates. The annotation and review of the phylogenetic trees were completed by iTOL (https://itol.embl.de/). The ring visible on the exterior of the tree indicates MICs of LL-37.

### Preparation of LL37^I20M/V21R^ or LL-37–loaded CS-NPs

Given the challenges associated with unstable properties and low oral bioavailability of peptides, we first prepared a chitosan-based delivery system for LL-37 ^I20M/V21R^ using the ionotropic gelation method (*30*). Chitosan (30 mg/ml) was dissolved in 1% (v/v) acetic acid solution (pH 5) under magnetic stirring. LL-37^I20M/V21R^ or LL-37 was gradually added to the chitosan solution and stirred continuously at 4°C for 24 hours, while blank CS-NPs were prepared without peptides. Subsequently, a TPP aqueous solution (1 mg/ml, pH 5) was introduced into the mixture at a 1:3 ratio and stirred at room temperature for an additional 4 hours. The nanoparticle suspension was purified by centrifugation at 14,000*g* and 4°C for 40 min, followed by multiple washes with deionized water. Last, the CS/LL37^I20M/V21R^-NPs and CS/LL37-NPs formulation were freeze-dried for 24 hours. After centrifuging of CS/LL37^I20M/V21R^-NPs or CS/LL37-NPs, the amount of free LL37^I20M/V21R^ or LL37 in supernatant was evaluated using a Micro BCA protein assay kit (Thermo Fisher Scientific, USA) at a wavelength of 565 nm. Encapsulation efficiency (EE) was calculated by the following formula, Encapsulation efficiency (%) = (total peptides − free peptides) / total peptides × 100.

To evaluate the pH stability of the nanoparticles, 3 mg of CS/LL37^I20M/V21R^-NPs or CS/LL37-NPs were incubated in 2 ml of PBS at pH 2, 4, 6, 8, and 10 for 30 min at room temperature. The samples were centrifuged at 5000 rpm for 10 min, and the supernatant was collected. Retention rate (%) = (Total peptides − free peptides) / total peptides ×100.

### Mice colonization assay

All animal experiments were conducted with the approval of the Ethics Committee of the Experimental Animal at Yangzhou University (202309580). To evaluate the ability of *C. jejuni* to evade LL-37 and establish colonization, 6-week-old C57BL/6 mice (20 ± 2 g) were obtained from the Centre for Comparative Medicine at Yangzhou University. Mice were pretreated with 200 μl of sodium bicarbonate solution (50 mg/ml), followed 15 to 30 min later by orogastric inoculation with 1 × 10^8^ CFU of *C. jejuni* 81-176 or P116B strains on day 0. Mice were randomly assigned to experimental groups, and the sample size was determined on the basis of prior experiments. On day 4 postinfection, when cecal bacterial loads had stabilized, each group of mice (*n* = 6) received a single oral dose of 200 μl of either PBS (control), CS/LL-37-NPs [containing LL-37 (11 μg/ml) for strain 81-176 or for P116B (88 μg/ml)], CS/LL-37^I20M/V21R^-NPs [containing LL-37^I20M/V21R^ (2.75 μg/ml), or blank CS-NPs, all formulated in PBS. After 24 hours, the mice were euthanized by cervical dislocation, and their cecal contents were carefully collected into 2.0-ml sterile plastic tubes containing 1.0 ml of PBS. The tubes were weighed before and after transferring the contents to determine the sample weight. Serial dilutions of the homogenized cecal contents were plated onto selective agar containing amphotericin B and cycloheximide and incubated for 48 hours at 42°C under microaerophilic conditions (*86*). Bacterial colonies were counted and normalized to the weight of each sample. All mice were included in the analysis; no animals were excluded.

### Statistical analysis

The data were analyzed using Prism GraphPad software (version 6.01) by Student’s *t* test. A statistical analysis of survival curves was performed using the log-rank test. Statistically significant differences are: *P < 0.05, **P < 0.01, ***P < 0.001. Statistically not significant (ns) was denoted when the *P* value was >0.05. All samples and data points from all experiments were included in the analysis; no exclusions were made.
