# Supplementary material for: Serine protease HtrA promotes Campylobacter jejuni intestinal colonization through degrading antimicrobial peptide LL-37
Source: Sci Adv. 2026 May 20;12(21):eaee1996. doi: 10.1126/sciadv.aee1996 (PMC13189099; doi:10.1126/sciadv.aee1996)
Supplement: Supplementary file 1 — Figs. S1 to S8 Tables S1 to S18 Legend for data S1 Legend for movie S1 [file sciadv.aee1996_sm.pdf]

Science Advances
AAAS

Supplementary Materials for

Serine protease HtrA promotes *Campylobacter jejuni* intestinal colonization through degrading antimicrobial peptide LL-37

Xiaofei Li *et al.*

Corresponding author: Jinlin Huang, jinlin@yzu.edu.cn; Ozan Gundogdu, ozan.gundogdu@lshtm.ac.uk

Sci. Adv. **12**, eaee1996 (2026)
DOI: 10.1126/sciadv.aee1996

The PDF file includes:

Figs. S1 to S8
Tables S1 to S18
Legend for data S1
Legend for movie S1

Other Supplementary Material for this manuscript includes the following:

Data S1
Movie S1

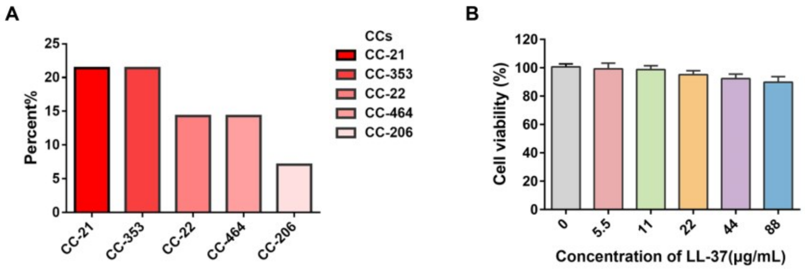

Fig. S1. Bactericidal activity of LL-37 against *C. jejuni*. (**A**) The distribution of clonal complexes (CCs) among clinical isolates with MIC values exceeding 88 μg/mL. (**B**) Effects of LL-37 on Caco-2 cell viability assessed with the MTT assay. Data are expressed as the mean ± SE from three separate experiments.

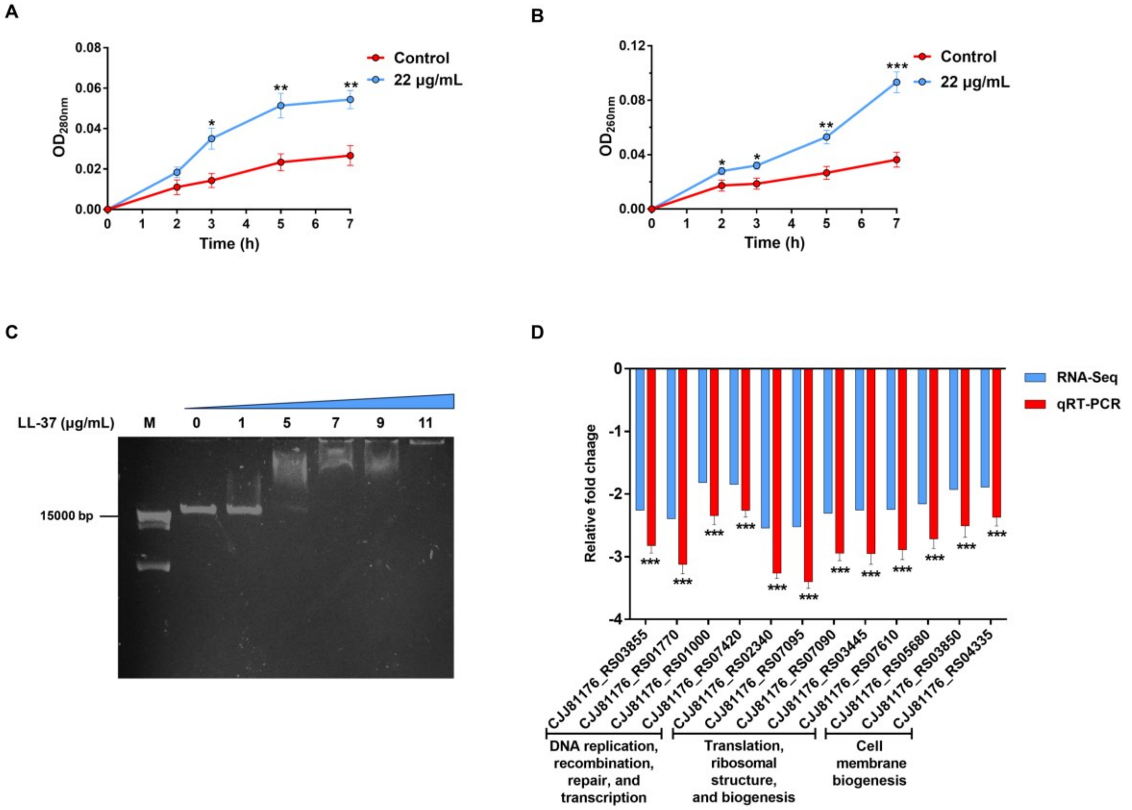

Fig. S2. LL-37 has an effect on membrane integrity. (**A** and **B**) Leakage of intracellular nucleic acids (OD_{260nm}) (**A**) and proteins (OD_{280nm}) (**B**) from cell suspensions of *C. jejuni* 81-176 strain exposed to 2 × MIC of LL-37. The non-treatment of 81-176 strain was used as a control. Error bars represented the standard deviation of independent replicates. Data were analyzed using Student's *t*-test (*$P < 0.05$, **$P < 0.01$, ***$P < 0.001$). (**C**) gel retardation assay. The interaction of LL-37 with *E. coli* ATCC25922 genomic DNA was evaluated by agarose gel retardation assay. The LL-37 concentration indicated in each lane represents a serial increase in concentration from 1 to 11 μg/mL**.** M, DL15000 DNA marker. (**D**) Validation of RNA-Seq results by qRT-PCR. Fold change comparison of RNA-Seq (blue bars) and quantitative real-time PCR (qRT-PCR, red bars) for randomly selected genes related to DNA replication, recombination, repair, transcription, translation, ribosome structure, biosynthesis, and cell membrane biogenesis. Error bars represented the standard deviation of independent replicates. Data were analyzed using Student's t-test (***$P < 0.001$).

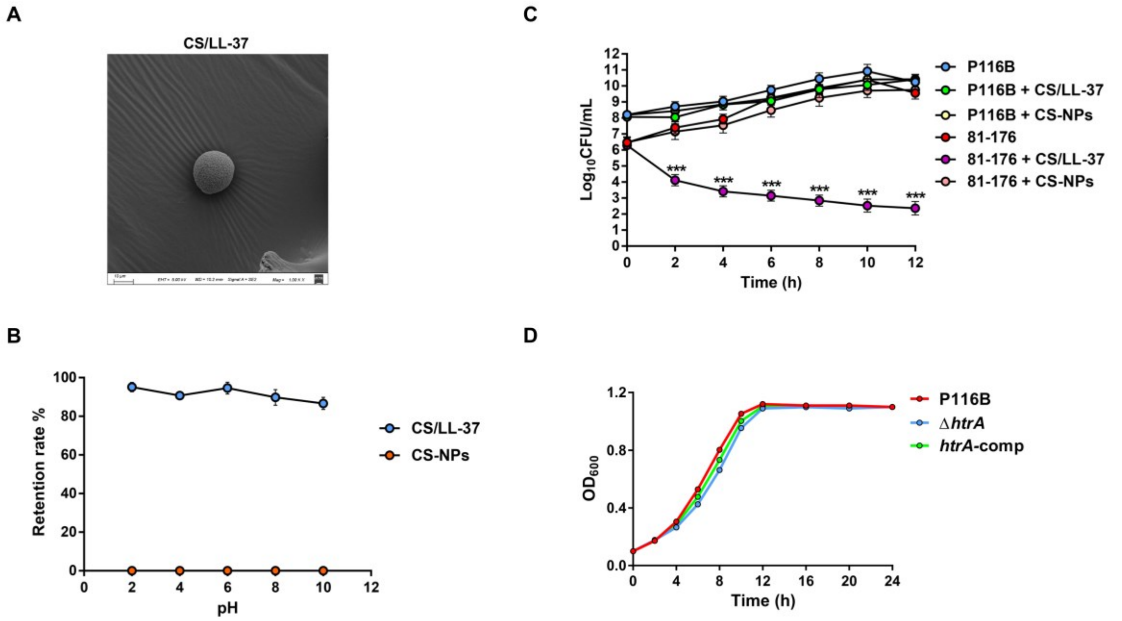

Fig. S3. Characterization of LL-37-loaded chitosan nanoparticles (CS-NPs). (**A**) SEM image of prepared CS/LL-37-NPs. (**B**) pH Stability of prepared CS/LL-37-NPs. CS/LL-37-NPs and blank CS-NPs were incubated at room temperature for 30 minutes in PBS with 2, 4, 6, 8, and 10 pH values. Error bars represented the standard deviation of independent replicates. (**C**) Kinetics of antibacterial potential of CS/LL-37-NPs against *C. jejuni* P116B and 81-176. Error bars represented the standard deviation of independent replicates. Data were analyzed using Student's *t*-test (*$P < 0.05$, **$P < 0.01$, ***$P < 0.001$). (**D**) Growth curve of *htrA* deletion and complementary strains. These data represent the mean from three separate experiments.

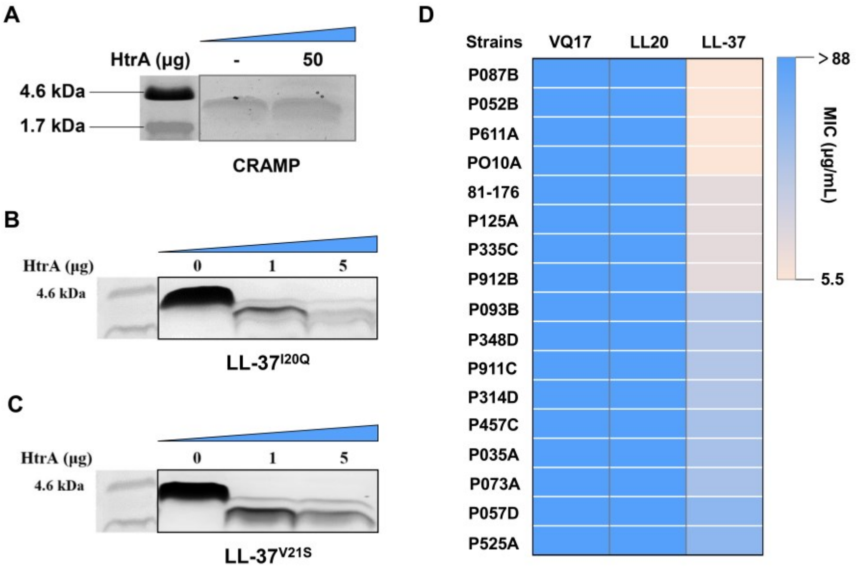

Fig. S4. Cleavage of CRAMP and LL-37 mutants by HtrA and antimicrobial spectrum of LL-37 cleavage products. (**A**) SDS-PAGE analysis of CRAMP cleavage after incubation with HtrA for 2 h. The gel was visualized using Coomassie blue staining. (**B** and **C**) SDS-PAGE image of LL-37^{I20Q} (**B**) and LL-37^{V21S} (**C**). The single-point mutants LL-37^{V21S} and LL-37^{I20Q} (1 µg) were incubated with HtrA for 2 h. (**D**) MIC heatmap of LL20 and VQ17 peptides against 17 *C. jejuni* clinical isolates, which were randomly selected for testing based on the MIC values of LL-37. MIC values are color-coded, and the color key is shown on the right in this figure. The peptides' names are shown at the top.

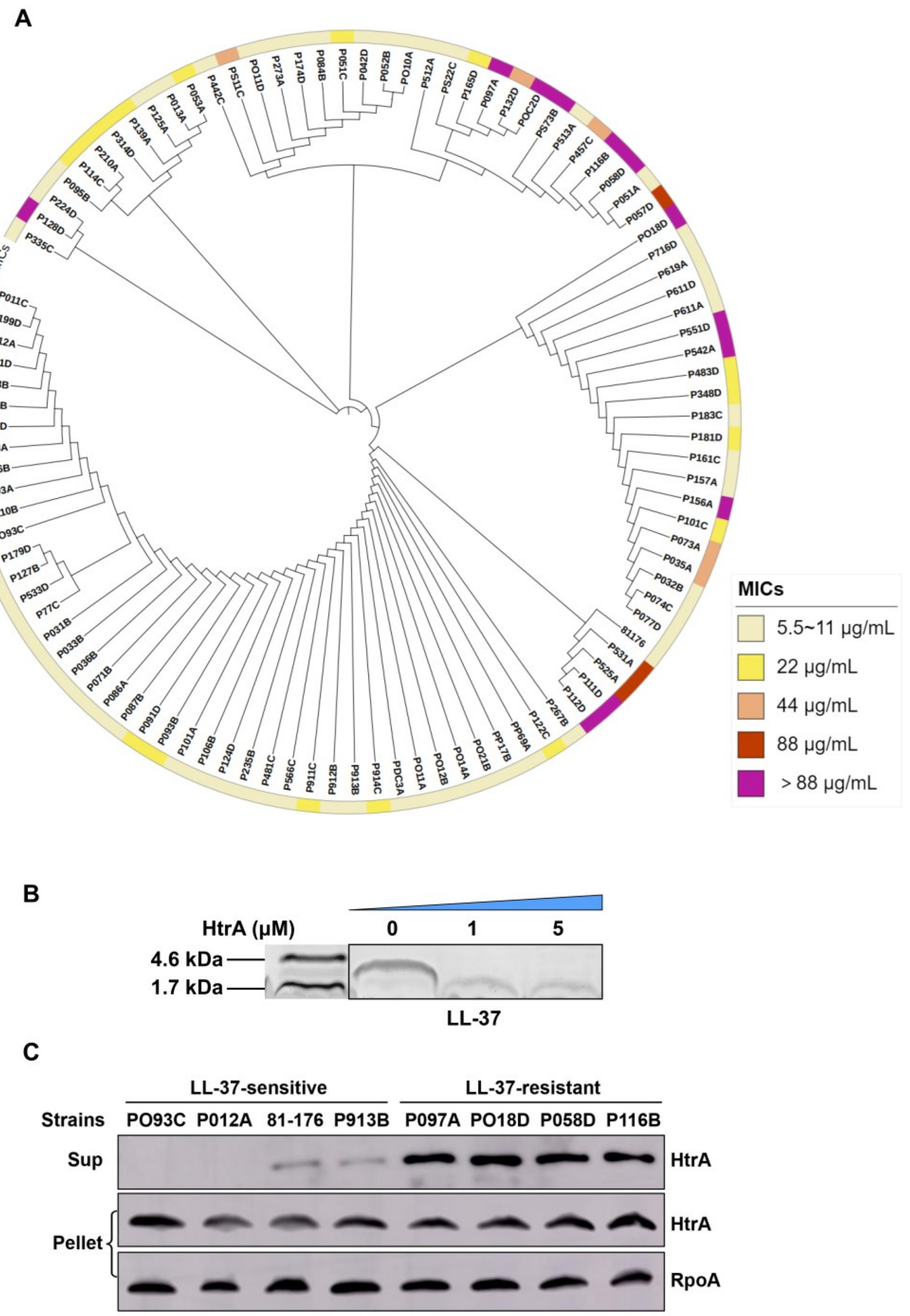

Fig. S5. The HtrA-mediated LL-37 cleavage is conserved in *C. jejuni*. (**A**) The phylogenetic tree of the HtrA from 102 *C. jejuni* clinical isolates. The tree was constructed with MEGA 6.0 using the maximum likelihood (ML) method. Bootstrap values in percentages are 1000 replicates. iTOL was used to annotate and review the phylogenetic tree. The ring visible on the exterior of the tree indicates MICs of LL-37. (**B**) SDS-PAGE analysis of LL-37 cleavage after incubation with HtrA from 81-176 strain for 2 h. (**C**) Western blot analysis of HtrA in supernatants (Sup) and pellets from LL-37-sensitive strains and LL-37-resistant strains exposed to LL-37 (5.5 μg/mL). RpoA was used as a loading control.

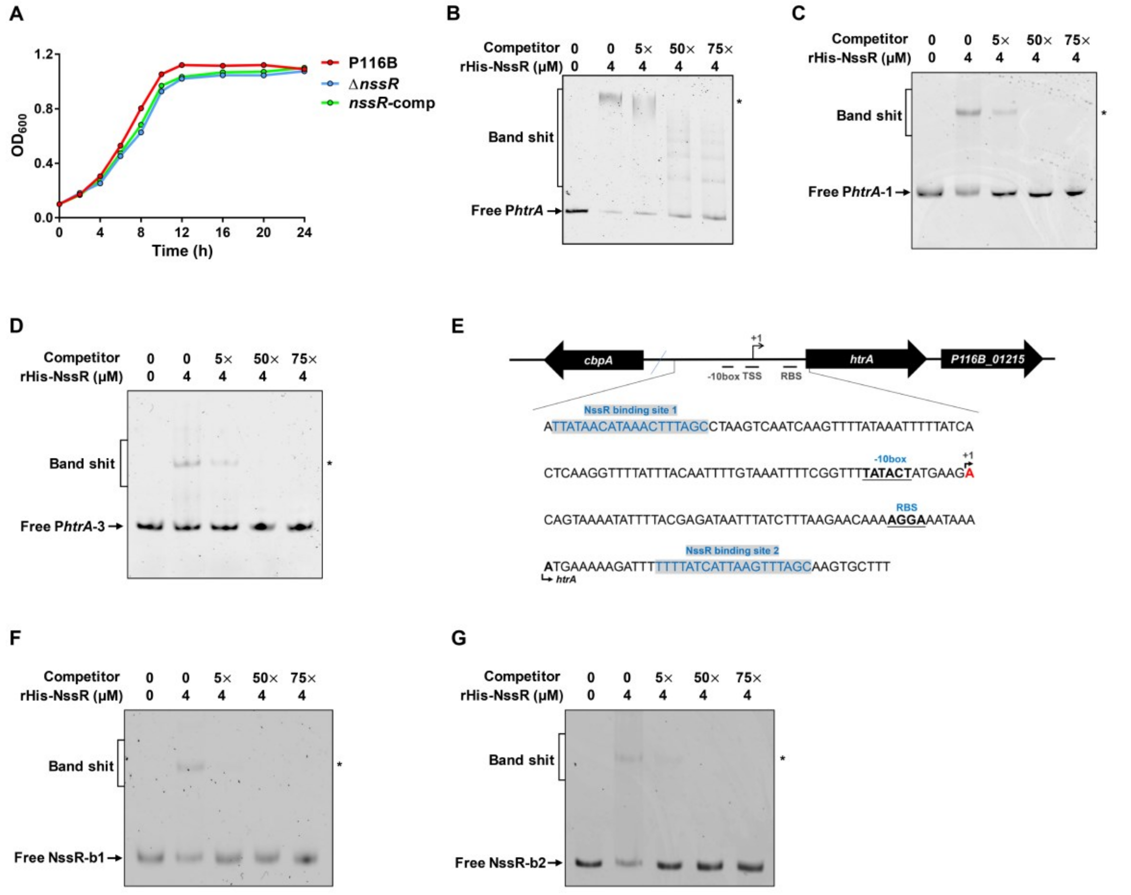

Fig. S6. Two NssR-binding sites were identified on P*htrA* promoter. (**A**) Growth curve of *nssR* deletion and complementary strains. These data represent as mean from three separate experiments. (**B-D**) A competitive EMSA experiment for P*htrA* (**B**), P*htrA*-1 (**C**), and P*htrA*-3 (**D**). The unlabeled DNA probe was used as a competitor. DNA-protein complexes are indicated by an asterisk. (**E**) Diagram showing the promoter region of the *htrA* gene. The ribosome-binding site (RBS) was underlined, and the transcription start site (TSS) was labeled as +1 and marked in red. The -10 box was located directly upstream of the transcriptional start site +1A. The NssR-binding sites were marked in gray. (**F and G**) A competitive EMSA experiment for NssR-b1 (**F**) and NssR-b2 (**G**). The unlabeled DNA probe was used as a competitor. DNA-protein complexes are indicated by an asterisk.

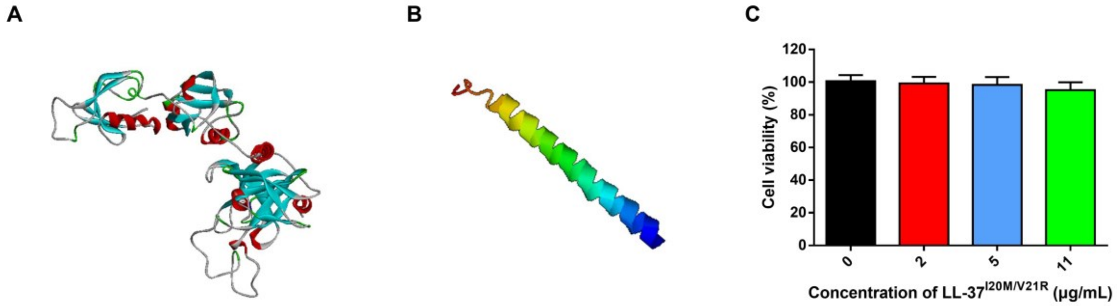

Fig. S7. Structure of HtrA and LL-37. (**A** and **B**) Protein structure of HtrA (**A**) and LL-37 (PDB: 5NMN) (**B**). The structure of HtrA was predicted using AlphaFold2. (**C**) Effects of LL-$37^{I20M/V21R}$ on Caco-2 cell viability assessed with the MTT assay. Data are expressed as the mean ± SE from three separate experiments. Statistically significant differences in relative viability between certain LL-$37^{I20M/V21R}$ doses and control are indicated by an asterisk (*) ($p < 0.05$).

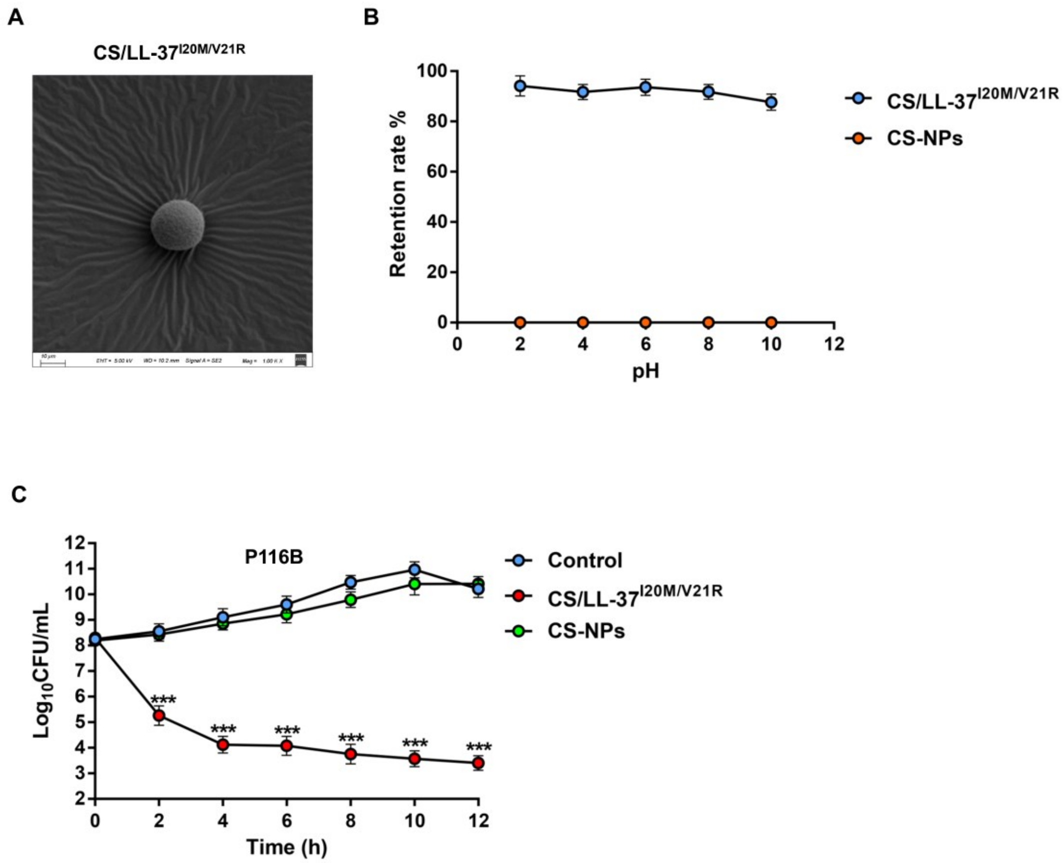

Fig. S8. Characterization of LL-37$^{I20M/V21R}$-loaded chitosan nanoparticles (CS-NPs). (**A**) SEM images of prepared CS/LL-37$^{I20M/V21R}$-NPs. (**B**) pH Stability of prepared CS/LL-37$^{I20M/V21R}$-NPs. CS/LL-37$^{I20M/V21R}$-NPs, and blank CS-NPs were incubated at room temperature for 30 minutes in PBS with 2, 4, 6, 8, and 10 pH values. Data are displayed as the mean ± SEM from three independent repeats. (**C**) Kinetics of antibacterial potential of LL-37$^{I20M/V21R}$-NPs against *C. jejuni* P116B. Results are reported as mean ± SEM from three individual tests. Data were analyzed using Student's *t*-test (*$P < 0.05$, **$P < 0.01$, ***$P < 0.001$).

Table S1. MIC values of LL-37 and LL-37$^{I20M/V21R}$ against 102 clinical *C. jejuni* isolates.

Name	Host	ST	CC	MIC (µg/mL)	
				LL-37	LL-37$^{I20M/V21R}$
P116B	Human	760	CC21	＞88	2.75
PS73B	Human	760	CC21	＞88	5.5
P058D	Human	1811	CC21	＞88	5.5
P111D	Human	22	CC22	＞88	5.5
P112D	Human	22	CC22	＞88	11
P551D	Human	2132	CC353	＞88	5.5
P097A	Human	6499	CC353	＞88	5.5
P542A	Human	5	CC353	＞88	11
PO18D	Human	464	CC464	＞88	2.75
P156A	Human	464	CC464	＞88	5.5
POC2D	Human	2282	CC206	＞88	2.75
P128D	Human	11903		＞88	22
P083A	Human	2276		＞88	11
P081D	Human	2276		＞88	11
P531A	Human	3652	CC22	88	5.5
P525A	Human	3652	CC22	88	5.5
P057D	Human	1811	CC21	88	5.5
PS11C	Human	12363		44	5.5
P457C	Human	50	CC21	44	5.5
P132D	Human	2282	CC206	44	2.75
P073A	Human	5	CC353	44	5.5
P035A	Human	5	CC353	44	5.5
P914C	Human	11160	CC607	22	2.75
P911C	Human	11160	CC607	22	11
P483D	Human	4331	CC607	22	5.5
P348D	Human	4331	CC607	22	5.5
P314D	Human	45	CC45	22	11
P210A	Human	4250		22	11
P181D	Human	4331	CC607	22	2.75
P165D	Human	4240		22	11
P139A	Human	137	CC45	22	5.5
P122C	Human	2895	CC574	22	5.5
P114C	Human	4250		22	11
P110B	Human	4246	CC257	22	2.75
P101C	Human	2274		22	11
P093B	Human	4261	CC52	22	5.5
P091D	Human	9961		22	2.75
P078B	Human	4246	CC257	22	5.5
P053A	Human	137	CC45	22	5.5
P051C	Human	21	CC21	22	5.5
PS22C	Human	4240		11	5.5
PP17B	Human	2133		11	5.5
PO14A	Human	2842	CC353	11	5.5
PO11D	Human	51	CC443	11	2.75
P913B	Human	11160	CC607	11	5.5
P912B	Human	11160	CC607	11	11

P77C	Human	4327		11	5.5
P619A	Human	4326	CC353	11	11
P481C	Human	1232	CC353	11	5.5
P335C	Human	45	CC45	11	5.5
P273A	Human	51	CC443	11	5.5
P183C	Human	2274		11	5.5
P174D	Human	463	CC443	11	5.5
P127B	Human	2328		11	11
P125A	Human	45	CC45	11	5.5
P096B	Human	2276		11	5.5
P086A	Human	6702	CC464	11	11
P084B	Human	463	CC443	11	5.5
P074C	Human	12367		11	11
P071B	Human	354	CC354	11	5.5
P051A	Human	4262		11	5.5
P033B	Human	918	CC48	11	11
P013A	Human	3930		11	2.75
81176	Human	604	CC42	11	5.5
PP69A	Human	8727	CC464	5.5	5.5
PO93C	Human	990	CC257	5.5	5.5
PO21B	Human	4324		5.5	5.5
PO12B	Human	4324		5.5	5.5
PO11A	Human	2842	CC353	5.5	5.5
PO10A	Human	51	CC443	5.5	5.5
PDC3A	Human	354	CC354	5.5	5.5
P716D	Human	3906	CC464	5.5	5.5
P611D	Human	4346		5.5	2.75
P611A	Human	3906	CC464	5.5	5.5
P566C	Human	2842	CC353	5.5	5.5
P533D	Human	4325		5.5	2.75
P513A	Human	4262		5.5	5.5
P512A	Human	6606		5.5	5.5
P442C	Human	1269	CC1034	5.5	5.5
P267B	Human	2274		5.5	5.5
P235B	Human	4248		5.5	5.5
P224D	Human	692	CC692	5.5	2.75
P199D	Human	4344	CC574	5.5	5.5
P179D	Human	4327		5.5	2.75
P161C	Human	3906	CC464	5.5	2.75
P157A	Human	9482	CC464	5.5	5.5
P124D	Human	2842	CC353	5.5	2.75
P106B	Human	1953	CC353	5.5	5.5
P103A	Human	11687		5.5	5.5
P101A	Human	1232	CC353	5.5	5.5
P095B	Human	3578		5.5	2.75
P087B	Human	6702	CC464	5.5	5.5
P077D	Human	12367		5.5	5.5
P073B	Human	4258	CC353	5.5	5.5
P052B	Human	51	CC443	5.5	2.75

P042D	Human	4266		5.5	5.5
P036B	Human	1953	CC353	5.5	5.5
P032B	Human	5	CC353	5.5	5.5
P031B	Human	6702	CC464	5.5	5.5
P021D	Human	305	CC574	5.5	2.75
P012A	Human	305	CC574	5.5	5.5
P011C	Human	305	CC574	5.5	5.5

Table S2. MIC and MBC values of LL-37 against *C. jejuni* 81-176 strain. The Scrm was a negative control.

**Strain**	**LL-37**		**Scrm**
	**MIC (μg/mL)**	**MBC (μg/mL)**	**MIC (μg/mL)**
81-176	11	22	＞88

Table S3. Genes differentially expressed in *C. jejuni* 81-176 in response to LL-37 exposure. Genes with at least a 1.5-fold decrease and a p-value ≤ 0.05 were considered significant.

Gene_ID	Log2(Fold Change)	P value	description
DNA replication, recombination, and repair			
CJJ81176_RS03855	-1.173	2.28E-07	Holliday junction branch migration protein RuvA
CJJ81176_RS01000	-0.860	0.002122	AAA family ATPase
CJJ81176_RS01050	-0.855	0.002198	DNA adenine methylase
CJJ81176_RS05625	-0.849	3.35E-07	DNA polymerase III subunit gamma/tau
CJJ81176_RS04370	-0.650	0.000113	HU family DNA-binding protein
CJJ81176_RS05110	-0.631	7.55E-06	endonuclease MutS2
CJJ81176_RS02855	-0.627	0.001392	NAD-dependent DNA ligase LigA
CJJ81176_RS00005	-0.626	0.000175	chromosomal replication initiator protein DnaA
Transcription			
CJJ81176_RS01770	-1.258	1.47E-07	multidrug efflux system transcriptional regulator CmeR
CJJ81176_RS07420	-0.884	3.24E-06	helix-turn-helix domain-containing protein
CJJ81176_RS05620	-0.808	1.76E-06	transcription termination factor Rho
CJJ81176_RS05985	-0.649	0.001376	heat shock transcriptional regulator HspR
CJJ81176_RS07630	-0.631	0.002278	DNA-directed RNA polymerase subunit alpha
CJJ81176_RS04855	-0.610	0.000157	RNA polymerase sigma factor RpoD
Translation, ribosomal structure, and biogenesis			
CJJ81176_RS02340	-1.346	5.25E-13	50S ribosomal protein L10
CJJ81176_RS07095	-1.334	7.69E-10	50S ribosomal protein L13
CJJ81176_RS07090	-1.206	1.44E-10	30S ribosomal protein S9
CJJ81176_RS03445	-1.174	0.000112	ribosome maturation factor RimM
CJJ81176_RS07610	-1.166	1.01E-08	50S ribosomal protein L36
CJJ81176_RS05205	-1.156	1.15E-09	30S ribosomal protein S18
CJJ81176_RS03435	-1.050	4.57E-07	30S ribosomal protein S16
CJJ81176_RS08135	-1.009	3.68E-08	50S ribosomal protein L15
CJJ81176_RS07615	-0.929	1.82E-07	30S ribosomal protein S13
CJJ81176_RS08150	-0.924	2.76E-08	50S ribosomal protein L6
CJJ81176_RS07605	-0.891	0.004171	translation initiation factor IF-1
CJJ81176_RS02345	-0.886	1.35E-07	50S ribosomal protein L7/L12
CJJ81176_RS08140	-0.840	7.67E-06	30S ribosomal protein S5
CJJ81176_RS07710	-0.839	6.39E-05	30S ribosomal protein S20
CJJ81176_RS08145	-0.837	4.35E-06	50S ribosomal protein L18
CJJ81176_RS00810	-0.819	2.50E-05	50S ribosomal protein L31
CJJ81176_RS03405	-0.819	1.04E-05	glycine--tRNA ligase subunit alpha
CJJ81176_RS08220	-0.805	0.0027749	50S ribosomal protein L4
CJJ81176_RS08175	-0.799	1.05E-05	50S ribosomal protein L14
CJJ81176_RS05815	-0.749	0.0005609	Asp-tRNA(Asn)/Glu-tRNA(Gln) amidotransferase subunit GatB
CJJ81176_RS02185	-0.748	0.0002423	50S ribosomal protein L28
CJJ81176_RS06645	-0.745	0.0002362	selenocysteine-specific translation elongation factor
CJJ81176_RS07625	-0.730	1.80E-05	30S ribosomal protein S4
CJJ81176_RS08215	-0.721	0.0059422	50S ribosomal protein L23

CJJ81176_RS03455	-0.705	5.24E-05	50S ribosomal protein L19
CJJ81176_RS04675	-0.702	5.68E-05	ribonuclease P protein component
CJJ81176_RS08170	-0.696	0.0001260	50S ribosomal protein L24
CJJ81176_RS04030	-0.687	9.45E-06	methionine--tRNA ligase
CJJ81176_RS04225	-0.680	0.0051805	30S ribosomal protein S15
CJJ81176_RS07620	-0.670	0.0002325	30S ribosomal protein S11
CJJ81176_RS07635	-0.652	0.0087558	50S ribosomal protein L17
CJJ81176_RS08155	-0.644	0.0030649	30S ribosomal protein S8
CJJ81176_RS00965	-0.644	5.32E-05	peptide deformylase
CJJ81176_RS04275	-0.621	0.0046086	30S ribosomal protein S1
CJJ81176_RS06100	-0.618	0.0016669	polyribonucleotide nucleotidyltransferase
CJJ81176_RS01780	-0.604	7.36E-05	30S ribosomal protein S21
Cell membrane biogenesis			
CJJ81176_RS05680	-1.108	6.82E-09	DUF3373 family protein
CJJ81176_RS03850	-0.947	8.47E-08	D-alanine--D-alanine ligase
CJJ81176_RS04335	-0.918	3.86E-06	alanine racemase
CJJ81176_RS03140	-0.842	7.15E-07	lytic transglycosylase domain-containing protein
CJJ81176_RS05275	-0.721	1.68E-05	polymer-forming cytoskeletal protein
CJJ81176_RS04150	-0.721	0.0140536	bifunctional anthranilate synthase component I family protein/aminotransferase class IV
CJJ81176_RS05550	-0.713	0.0015471	UDP-N-acetylglucosamine 2-epimerase (hydrolyzing)
CJJ81176_RS06795	-0.709	0.0001139	capsule polysaccharide modification protein KpsS
CJJ81176_RS07395	-0.668	0.0002875	lipocalin family protein
CJJ81176_RS06130	-0.662	0.0001051	group 1 major outer membrane porin protein PorA
CJJ81176_RS02105	-0.632	0.0005432	phospho-N-acetylmuramoyl-pentapeptide-transferase
CJJ81176_RS05545	-0.627	0.0066141	N-acetylneuraminate synthase
CJJ81176_RS03145	-0.624	0.0015010	septal ring lytic transglycosylase RlpA family protein
CJJ81176_RS06905	-0.614	0.0029172	glycosyltransferase family 8 protein
CJJ81176_RS02100	-0.612	0.0001526	UDP-N-acetylmuramoyl-L-alanine--D-glutamate ligase
CJJ81176_RS05320	-0.605	0.0260131	apolipoprotein N-acyltransferase

Table S4. Encapsulation efficiency of CS/LL-37 or CS/LL-37$^{I20M/V21R}$.

Nanoparticles	Encapsulation efficiency%
LL-37	89.38
LL-37$^{I20M/V21R}$	87.31
CS-NPs	-

Table S5. Genes differentially expressed in *C. jejuni* P116B in response to LL-37 exposure. Genes with Log2 (Fold Change) ≥1.0 and a p-value ≤ 0.05 were considered significant.

Gene_ID	Log2(FC)	P value	description
P116B_00744	5.114	0.0058771	IS200/IS605 family transposase ISCco1
P116B_01428	4.611	7.52E-78	catalase
P116B_00041	4.573	1.57E-08	cytochrome C
P116B_01549	4.412	1.15E-31	inner membrane protein
P116B_01159	4.397	1.02E-22	outer membrane protein
P116B_00737	4.324	0.0082146	outer-membrane protein
P116B_01674	4.308	3.76E-24	ExbB/TolQ family transport protein
P116B_01550	4.245	2.17E-34	Putative sulfur carrier protein YedF
P116B_01429	4.041	3.53E-65	ankyrin repeat-containing protein
P116B_01158	3.929	4.34E-19	periplasmic protein
P116B_01704	3.895	3.85E-21	iron permease
P116B_01705	3.555	3.86E-25	34 kDa membrane antigen
P116B_00286	3.422	1.32E-71	3-methyl-2-oxobutanoate hydroxymethyltransferase
P116B_01707	3.343	1.73E-24	ABC transporter permease
P116B_01394	3.327	4.06E-09	enterochelin uptake permease
P116B_01397	3.3	7.01E-30	enterochelin uptake substrate-binding protein
P116B_00291	3.298	6.10E-42	molybdenum ABC transporter substrate-binding lipoprotein
P116B_00253	3.221	2.87E-44	zinc transporter ZupT
P116B_01319	3.192	6.44E-45	hypothetical protein
P116B_01708	3.174	1.18E-29	integral membrane protein
P116B_00745	3.16	8.40E-38	TonB transport protein
P116B_01229	3.15	2.93E-16	MFS transport protein
P116B_01656	3.102	7.99E-16	hemin uptake system outer membrane receptor
P116B_00285	3.071	4.95E-36	pantothenate synthetase
P116B_01710	3.006	1.00E-22	thiredoxin
P116B_00310	2.999	1.24E-19	peroxide stress regulator
P116B_00365	2.994	3.00E-24	sulfoxide reductase catalytic subunit
P116B_00024	2.967	2.24E-09	sodium:dicarboxylate family transmembrane symporter
P116B_00900	2.946	1.32E-40	acyl-CoA thioesterase
P116B_01709	2.92	1.26E-41	ABC transporter ATP-binding protein
P116B_01208	2.864	3.24E-30	chaperone GroEL
P116B_00059	2.863	1.99E-14	periplasmic protein
P116B_00749	2.861	4.99E-19	chaperone DnaK
P116B_01706	2.821	8.93E-20	integral membrane protein
P116B_00433	2.81	9.41E-16	ferric enterobactin uptake receptor
P116B_00054	2.78	0.0090927	hypothetical protein
P116B_00353	2.754	1.70E-52	multidrug efflux pump protein CmeA
P116B_00060	2.753	1.23E-14	peptidase C39 family protein
P116B_01621	2.723	1.36E-17	peptide ABC transporter ATP-binding protein
P116B_00484	2.72	1.84E-21	proline dehydrogenase/delta-1-pyrroline-5-carboxylate dehydrogenase
P116B_00231	2.719	5.66E-21	cysteine desulfurase
P116B_00400	2.708	1.26E-10	oxidoreductase subunit
P116B_00748	2.677	9.26E-09	heat shock protein GrpE
P116B_01427	2.612	0.0035858	hypothetical protein

P116B_00284	2.578	4.01E-40	aspartate 1-decarboxylase
P116B_01711	2.546	5.80E-18	lipoprotein thiredoxin
P116B_00401	2.546	4.99E-08	GMC oxidoreductase subunit
P116B_01207	2.517	8.70E-19	co-chaperonin GroES
P116B_01128	2.503	0.016046	glycosyltransferase
P116B_00482	2.48	1.02E-15	amidohydrolase
P116B_01439	2.48	0.0019566	hypothetical protein
P116B_00364	2.439	0.0029688	ferric reductase-like transmembrane protein
P116B_00237	2.426	0.0096108	hypothetical protein
P116B_00626	2.387	4.93E-28	lipoprotein
P116B_00481	2.383	1.08E-10	sugar transporter
P116B_00502	2.381	4.33E-11	chaperone protein ClpB
P116B_00612	2.353	1.90E-09	phosphate ABC transporter substrate-binding protein
P116B_00722	2.337	4.78E-16	substrate-binding protein
P116B_00480	2.325	2.90E-12	short chain dehydrogenase
P116B_00991	2.294	5.05E-10	periplasmic protein
P116B_01285	2.287	0.0025782	hypothetical protein
P116B_00483	2.241	3.46E-16	L-rhamnose mutarotase
P116B_00963	2.236	1.10E-09	outer-membrane protein
P116B_00718	2.224	1.89E-16	integral membrane zinc-metalloprotease
P116B_00723	2.213	3.98E-15	periplasmic protein
P116B_01557	2.197	4.41E-09	formate dehydrogenase accessory protein
P116B_00039	2.157	3.78E-21	efflux protein
P116B_00890	2.156	8.42E-17	tRNA (cytidine(34)-2'-O)-methyltransferase
P116B_00959	2.154	0.0121459	hypothetical protein
P116B_01712	2.149	5.94E-15	periplasmic protein
P116B_00548	2.143	1.05E-15	dicarboxylate carrier protein MatC
P116B_00747	2.131	6.80E-08	heat-inducible transcription repressor
P116B_00476	2.107	4.02E-11	dihydrodipicolinate synthase
P116B_00939	2.103	4.63E-17	lipoprotein
P116B_00923	2.1	0.0006269	integral membrane protein
P116B_00230	2.089	1.11E-15	nitrogen fixation protein NifU
P116B_00341	2.077	4.71E-18	ferredoxin
P116B_00479	2.074	3.26E-08	Inner membrane transport protein RhmT
P116B_01172	2.059	2.48E-16	cyclopropane-fatty-acyl-phospholipid synthase
P116B_00672	2.057	0.002447	Potassium-transporting ATPase KdpC subunit
P116B_00322	2.049	1.06E-14	alkyl hydroperoxide reductase
P116B_00478	2.048	2.92E-12	Altronate dehydratase
P116B_00352	2.036	3.25E-27	multidrug efflux pump protein CmeB
P116B_01449	2.032	3.81E-13	periplasmic protein
P116B_00455	2.031	6.79E-15	group 3 truncated hemoglobin
P116B_00443	2.029	1.14E-13	phosphomethylpyrimidine synthase
P116B_00970	2.027	1.94E-07	amino acid transporter substrate-binding protein
P116B_01307	1.999	2.23E-10	hypothetical protein
P116B_00495	1.978	1.47E-10	Ammonium transporter
P116B_00477	1.972	3.31E-13	Altronate dehydratase
P116B_00351	1.968	5.97E-21	multidrug efflux pump protein CmeC
P116B_00849	1.945	2.51E-10	hypothetical protein
P116B_00145	1.939	1.34E-15	thioredoxin reductase
P116B_00278	1.939	3.20E-17	major antigenic peptide PEB3

P116B_00174	1.932	2.51E-08	iron-uptake ABC transporter substrate-binding protein
P116B_00259	1.922	2.33E-26	branched-chain amino acid aminotransferase
P116B_00717	1.91	2.66E-15	DNA methylase
P116B_01283	1.901	0.0029352	hypothetical protein
P116B_01660	1.896	7.33E-11	radical SAM domain-containing protein
P116B_00252	1.887	1.34E-07	methyl-accepting chemotaxis signal transduction protein
P116B_01211	1.851	1.35E-09	iron-binding protein
P116B_00227	1.835	5.95E-19	integral membrane protein
P116B_00044	1.833	0.0021558	hypothetical protein
P116B_01659	1.831	1.18E-05	hemin uptake system substrate-binding protein
P116B_01162	1.801	2.72E-07	multidrug resistance protein
P116B_01676	1.791	0.000345	TonB transport protein
P116B_00769	1.774	7.27E-09	2-Cys peroxiredoxin
P116B_00724	1.77	1.71E-06	type I phosphodiesterase/nucleotide pyrophosphatase
P116B_01325	1.768	3.25E-07	K+ uptake protein
P116B_00738	1.752	0.0041066	outer-membrane protein
P116B_00887	1.743	8.02E-07	amino acid ABC transporter permease
P116B_01320	1.707	3.31E-07	hypothetical protein
P116B_00093	1.698	1.16E-12	50S ribosomal protein L21
P116B_00456	1.696	0.0132929	transcriptional regulator
P116B_01735	1.683	7.84E-20	DNA topoisomerase I
P116B_01396	1.683	2.25E-05	enterochelin uptake ATP-binding protein
P116B_01437	1.669	4.78E-10	adenylosuccinate lyase
P116B_00435	1.665	7.04E-15	ferric enterobactin uptake receptor
P116B_00289	1.646	1.36E-06	molybdenum ABC transporter permease
P116B_00549	1.639	6.72E-10	amidohydrolase family protein
P116B_01657	1.634	7.66E-09	hemin uptake ABC transporter permease
P116B_01743	1.622	2.65E-12	30S ribosomal protein S14
P116B_01152	1.621	2.29E-08	cation transport protein
P116B_01565	1.592	1.19E-10	oxidoreductase
P116B_00732	1.588	7.19E-08	hypothetical protein
P116B_00728	1.582	1.35E-07	HAD-superfamily hydrolase
P116B_01324	1.575	1.22E-06	K+ uptake protein
P116B_00757	1.574	1.47E-16	phosphopantetheine adenylyltransferase
P116B_00673	1.565	1.57E-10	Sensor protein KdpD
P116B_00591	1.565	4.44E-10	periplasmic protein
P116B_01318	1.552	2.43E-12	hypothetical protein
P116B_00511	1.548	4.73E-14	chaperone protein HtpG
P116B_01216	1.546	5.69E-09	serine protease
P116B_00684	1.524	0.0220234	restriction/modification enzyme
P116B_00552	1.513	1.38E-07	pyridine nucleotide-disulfide oxidoreductase
P116B_00082	1.512	0.0003081	cytochrome bd oxidase subunit I
P116B_01728	1.49	1.58E-11	lipoprotein
P116B_00627	1.49	1.58E-11	lipoprotein
P116B_00295	1.49	1.73E-08	adenosylmethionine--8-amino-7-oxononanoate aminotransferase BioA
P116B_01761	1.458	1.59E-14	hypothetical protein
P116B_01770	1.453	3.24E-14	acetyltransferase
P116B_00746	1.443	1.33E-11	ferric enterobactin uptake receptor

P116B_01272	1.412	0.001301	hypothetical protein
P116B_01295	1.402	1.78E-09	hypothetical protein
P116B_00146	1.385	4.72E-12	thioredoxin
P116B_00083	1.379	1.19E-05	cytochrome bd oxidase subunit II
P116B_01287	1.371	1.64E-05	hypothetical protein
P116B_00897	1.364	1.97E-07	cysteine synthase
P116B_01556	1.353	0.0017627	regulatory protein
P116B_01627	1.351	0.0006274	single domain hemoglobin
P116B_00733	1.348	0.0015951	hypothetical protein
P116B_00727	1.344	0.0001911	ABC transporter ATP-binding protein
P116B_01275	1.326	0.0044858	hypothetical protein
P116B_00439	1.325	1.65E-10	50S ribosomal protein L28
P116B_00605	1.32	7.10E-06	secretion protein HlyD
P116B_00892	1.307	3.81E-11	periplasmic protein
P116B_01230	1.306	4.17E-06	hypothetical protein
P116B_00886	1.305	2.28E-05	hypothetical protein
P116B_01544	1.302	1.12E-07	hypothetical protein
P116B_00288	1.295	0.0001644	molybdenum ABC transporter ATP-binding protein
P116B_00859	1.295	8.31E-08	Thiol:disulfide interchange protein DsbL
P116B_00258	1.292	1.43E-09	transmembrane protein
P116B_00144	1.288	2.48E-05	TAT pathway signal sequence domain-containing protein
P116B_01763	1.282	0.0094024	hypothetical protein
P116B_00713	1.28	7.82E-07	DNA polymerase III subunit alpha
P116B_00671	1.276	4.30E-07	potassium-transporting ATPase subunit B
P116B_00185	1.275	2.99E-10	TerC family integral membrane protein
P116B_00681	1.274	8.70E-07	flagellar basal body L-ring protein
P116B_00209	1.272	3.37E-08	hypothetical protein
P116B_00888	1.269	1.01E-09	glutamine transporter ATP-binding protein
P116B_00202	1.249	0.0018286	citrate transporter
P116B_01771	1.238	0.0007125	3-isopropylmalate dehydratase small subunit
P116B_00520	1.235	1.64E-05	flagellar basal body rod protein FlgB
P116B_00177	1.225	6.01E-06	TonB-denpendent outer membrane receptor
P116B_01436	1.223	0.002311	O-acetylhomoserine (thiol)-lyase
P116B_01123	1.219	8.97E-09	hypothetical protein
P116B_01308	1.214	4.65E-07	hypothetical protein
P116B_00064	1.214	1.05E-06	integral membrane protein
P116B_00056	1.213	5.39E-09	lysine decarboxylase family protein
P116B_00211	1.206	2.00E-06	Temperature-sensitive hemagglutinin tsh autotransporter
P116B_01514	1.206	3.12E-06	flagellar biosynthesis protein FlgM
P116B_01779	1.205	0.0037532	homoserine O-succinyltransferase
P116B_01757	1.18	5.88E-07	30S ribosomal protein S10
P116B_01234	1.179	1.39E-09	excinuclease ABC subunit C
P116B_00038	1.174	6.58E-08	periplasmic protein
P116B_01778	1.172	4.07E-05	periplasmic protein
P116B_00432	1.158	2.46E-05	acetyl-CoA carboxylase carboxyl transferase subunit alpha
P116B_01212	1.153	0.0007497	hypothetical protein
P116B_01171	1.151	4.54E-07	30S ribosomal protein S2
P116B_01201	1.146	5.96E-09	exporting protein

P116B_00215	1.139	2.20E-10	N-acetyl-gamma-glutamyl-phosphate reductase
P116B_01525	1.125	3.40E-05	pyruvate-flavodoxin oxidoreductase
P116B_01205	1.124	5.32E-07	riboflavin synthase subunit alpha
P116B_01512	1.121	3.50E-06	flagellar basal body P-ring protein
P116B_01772	1.111	7.59E-07	3-isopropylmalate dehydratase large subunit
P116B_01085	1.108	6.64E-07	protein translocase subunit YajC
P116B_01594	1.108	5.35E-06	NADP-dependent alcohol dehydrogenase
P116B_00863	1.095	0.0001166	periplasmic protein
P116B_01294	1.094	1.15E-06	hypothetical protein
P116B_01780	1.086	0.0014466	O-acetylhomoserine (thiol)-lyase
P116B_01756	1.083	0.0001005	50S ribosomal protein L3
P116B_00168	1.08	0.0003073	superoxide dismutase
P116B_01161	1.075	4.57E-08	transcriptional regulator
P116B_00172	1.072	0.0041355	iron-uptake ABC transporter ATP-binding protein
P116B_00167	1.069	0.0035462	periplasmic protein
P116B_00121	1.068	0.0001934	hypothetical protein
P116B_01187	1.062	6.23E-05	S-ribosylhomocysteine lyase
P116B_00256	1.056	0.0022231	integral membrane protein
P116B_01248	1.051	0.0001757	chaperone protein DnaJ
P116B_00287	1.049	0.0022294	beta-lactamase
P116B_00647	1.049	0.0002645	OstA family protein
P116B_01622	1.04	0.0053091	peptide ABC transporter ATP-binding protein
P116B_00072	1.033	0.0063925	iron-binding protein
P116B_01567	1.033	0.0001017	molybdopterin converting factor subunit 2
P116B_00402	1.032	0.0004852	hypothetical protein
P116B_00096	1.032	4.04E-06	glutamate 5-kinase
P116B_00936	1.031	0.0001397	outer-membrane lipoprotein carrier protein
P116B_01742	1.03	4.95E-06	30S ribosomal protein S8
P116B_01282	1.021	4.35E-05	hypothetical protein
P116B_01563	1.021	0.0083537	hypothetical protein
P116B_01773	1.02	9.42E-05	3-isopropylmalate dehydrogenase
P116B_00429	1.016	0.0005221	transcriptional regulator
P116B_00615	1.016	0.0012634	phosphate ABC transporter ATP-binding protein
P116B_00826	1.009	6.41E-05	methylated-DNA--protein-cysteine methyltransferase
P116B_01055	1.008	0.0003589	hypothetical protein
P116B_01672	1.008	0.0012657	membrane protein
P116B_00164	1.006	1.45E-06	tRNA dimethylallyltransferase
P116B_00425	1.005	1.25E-05	pyridoxamine 5'-phosphate oxidase
P116B_01586	1.003	0.0018269	periplasmic protein

Table S6. KEGG annotation information of *htrA* gene.

Gene_ID	Name	Log2FC	P value	Pathway
P116B_01216	*htrA*	1.548	5.70E-09	map01503: Cationic antimicrobial peptide (CAMP) resistance

Abbreviations: FC, Fold Change.

Table S7. The MIC values of LL-37 against gene deletion and complementary strains.

Strains	MIC (μg/mL)
P116B	＞88
Δ*htrA*	5.5
htrA complemented	88
Δ*nssR*	11
nssR complemented	88

Table S8. HPLC-MS analysis of cleavage site of HtrA on LL-37.

Peptide	-10lgP	Mass	Length	Area
LGDFFRKSKEKIGKEFKRI	80.31	2325.332	19	2.00E+09
LLGDFFRKSKEKIGKEFKRI	77.35	2438.416	20	7.95E+11
VQRIKDFLRNLVPRTES	76.26	2070.1697	17	1.35E+11
RKSKEKIGKEFKRI	72.56	1746.0627	14	4.98E+06
GDFFRKSKEKIGKEFKRI	72.27	2212.248	18	6.27E+10
FFRKSKEKIGKEFKRI	71.67	2040.1996	16	7.51E+08
VQRIKDFLRNLVPR	68.69	1753.0475	14	4.03E+06
VQRIKDFLRNLVPRT	68.51	1854.0951	15	9.03E+07
KEKIGKEFKRIVQR	66.94	1758.0627	14	2.69E+07
DFFRKSKEKIGKEFKRI	65.1	2155.2266	17	2.15E+10
KDFLRNLVPRTES	63.78	1573.8575	13	4.23E+11
KEKIGKEFKRIVQRI	61.56	1871.1469	15	5.85E+10
KDFLRN(+.98)LVPRTES	61.4	1574.8416	13	8.19E+08
VQRIKDFLRNLVPRTE	60.83	1983.1377	16	5.73E+07
LLGDFFRKSKEKI	60.68	1579.9086	13	3.46E+09
DFLRNLVPRTES	60.64	1445.7627	12	2.33E+07
LLGDFFRKS	59.92	1081.592	9	1.14E+11
KEKIGKEFKRI	58.32	1374.8346	11	6.33E+10
FLRNLVPRTES	57.63	1330.7357	11	4.09E+08
SKEKIGKEFKRIVQRI	56.71	1958.1788	16	1.30E+08

-10lgP defines the confidence level of the peptide; Mass defines the molecular weight of the peptide; Length defines the length of the peptide; Area defines the peak area of the peptide.

Table S9. MIC values of LL20, VQ17 and LL-37 against 17 *C. jejuni* clinical isolates.

Strain	MIC (μg/mL)		
	LL20	VQ17	LL-37
P087B	＞88	＞88	5.5
P052B	＞88	＞88	5.5
P611A	＞88	＞88	5.5
PO10A	＞88	＞88	5.5
81-176	＞88	＞88	11
P125A	＞88	＞88	11
P335C	＞88	＞88	11
P912B	＞88	＞88	11
P093B	＞88	＞88	22
P348D	＞88	＞88	22
P911C	＞88	＞88	22
P314D	＞88	＞88	22
P457C	＞88	＞88	44
P035A	＞88	＞88	44
P073A	＞88	＞88	44
P057D	＞88	＞88	88
P525A	＞88	＞88	88

Table S10. ***htrA*** **expression in** ***C.jejuni*** **81-176 upon exposure to LL-37.**

Gene_ID	Log2(FC)	P value	description
CJJ81176_RS05975	1.981	3.243E-27	serine protease HtrA

Table S11. Four transcription factors with DNA-binding activity interacting with the *htrA* promoter were identified by DNA pull-down and mass spectrometry.

Protein ID	Protein	Description	Coverage (%)
EAQ73460.1	CosR	DNA-binding response regulator	34.98
EAQ73017.1	Fur	ferric uptake regulation protein	16.56
EAQ73447.2	NssR	transcriptional regulator, putative	11.44
EAQ72039.1	Cj1000	transcriptional regulator, LysR family	3.41

Table S12. MIC values of LL-37$^{I20Q/V21S}$ against *C. jejuni* strains.

Strains	MIC (μg/mL)	
	LL-37$^{I20Q/V21S}$	LL-37
P116B	＞88	＞88
81-176	22	11

Table S13. Predictions are ranked by Docking Score values and ordered from best to worst prediction.

Rank	Docking Score	Confidence Score
1	-212.32	0.78
2	-209.37	0.77
3	-204.23	0.75
4	-191.88	0.70
5	-187.83	0.68
6	-185.45	0.67
7	-183.84	0.66
8	-182.55	0.66
9	-182.51	0.66
10	-181.92	0.65

Table S14. ΔAffinity and ΔStability results for the double point mutant of LL-37.

Residue	Original	Mutated	ΔAffinity（kcal/mol）	ΔStability（kcal/mol）
20	ILE	MET	1.46	-3.18
21	VAL	ARG		
20	ILE	ARG	0.93	-6.7
21	VAL	MET		
20	ILE	ARG	0.78	-2.38
21	VAL	GLN		
20	ILE	PHE	0.75	-1.75
21	VAL	MET		
20	ILE	MET	0.72	-5.65
21	VAL	MET		
20	ILE	GLU	0.71	-3.58
21	VAL	MET		
20	ILE	LEU	0.59	-4.3
21	VAL	MET		
20	ILE	MET	0.58	-0.81
21	VAL	GLN		
20	ILE	ARG	0.3	-5.64
21	VAL	LEU		
20	ILE	MET	0.26	-4.16
21	VAL	LEU		
20	ILE	ARG	0.26	-3.97
21	VAL	ILE		
20	ILE	MET	0.08	-3.85
21	VAL	ILE		
20	ILE	LEU	0.02	-1.07
21	VAL	ILE		

20	ILE	ARG	-0.04	-4.53
21	VAL	GLU		
20	ILE	GLU	-0.05	-3.12
21	VAL	ILE		
20	ILE	GLN	-0.08	-0.31
21	VAL	MET		
20	ILE	LEU	-0.14	-0.77
21	VAL	GLU		
20	ILE	MET	-0.15	-3.54
21	VAL	GLU		
20	ILE	VAL	-0.18	-0.35
21	VAL	ILE		
20	ILE	ARG	-0.5	-1.16
21	VAL	HIE		
20	ILE	GLU	-6.35	-1.77
21	VAL	GLU		
20	ILE	GLN	-7.07	-0.95
21	VAL	GLU		

Table S15. Prediction of LL-37$^{I20M/V21R}$ biological activity, water solubility, and toxicity.

Peptide	Net charge	Molecular weight	PI	GRAVY	Toxicity
LL-37$^{I20M/V21R}$	+7	4.57 kDa	11.00	-1.03	Non-Toxin
LL-37	+6	4.49 kDa	10.61	-0.724	Non-Toxin

Abbreviations: PI, Isoelectric Point; GRAVY, Grand Average of Hydropathicity.

Table S16. MIC values of LL-37 and LL-37$^{I20M/V21R}$ against other enteric bacteria

Strains	MIC (μg/mL)	
	**LL-37$^{I20M/V21R}$**	**LL-37**
E. coli ATCC 25922	5.5	22
S. aureus USA300	11	22
Salmonella C50336	11	44
Lactobacillus Q2	＞88	＞88
Lactobacillus Q3	＞88	＞88
Clostridium butyricum Y2	＞88	＞88
Clostridium butyricum Y35	＞88	＞88

Table S17. Key resources table

REAGENT or RESOURCE	SOURCE	IDENTIFIER
**Strains**		
C. jejuni 81-176	ATCC	BAA-2151
C. jejuni P116B	Our laboratory	N/A
C. jejuni P116B: *htrA*-deletion strain (Δ*htrA* mutant strain)	This work	N/A
C. jejuni P116B: *htrA* complemented strain (*htrA*-comp strain)	This work	N/A
C. jejuni P116B: *nssR*-deletion strain (Δ*nssR* mutant strain)	This work	N/A
C. jejuni P116B: *nssR* complemented strain (*nssR*-comp strain)	This work	N/A
DH5α	TaKaRa	9057
BL21(DE3)	TIANGEN	CB105-02
E. coli ATCC 25922	Institutional collection	N/A
Salmonella C50336	Institutional collection	N/A
S. aureus USA300	Institutional collection	N/A
Lactobacillus Q2	Institutional collection	N/A
Lactobacillus Q3	Institutional collection	N/A
C. butyricum Y2	Institutional collection	N/A
C. butyricum Y35	Institutional collection	N/A
**Plasmids**		
pRK2013	ATCC	37159
pCold I	Our laboratory	N/A
pMD19T	TAKARA	3271-C1
pUOA18	Our laboratory	N/A
pCold I-*htrA* (P116B)	This work	N/A
pCold I-$htrA^{S225A}$ (P116B)	This work	N/A
pCold I-*htrA* (81-176)	This work	N/A
pCold I-*nssR* (P116B)	This work	N/A
pMD19T-$htrA\text{-}kan^{r}$	This work	N/A
pMD19T-$nssR\text{-}kan^{r}$	This work	N/A
pUOA18-P*metK*-*htrA*	This work	N/A
pUOA18-P*metK*-*nssR*	This work	N/A
**peptides**		
LL-37 (LLGDFFRKSKEKIGKEFKRIVQRIKDFLRNLVPRTES)	AnHui JYHX CO., LTD	This work
LL-37^{I20Q} (LLGDFFRKSKEKIGKEFKRQVQRIKDFLRNLVPRTES)	AnHui JYHX CO., LTD	This work
LL-37^{V21S} (LLGDFFRKSKEKIGKEFKRISQRIKDFLRNLVPRTES)	AnHui JYHX CO., LTD	This work

LL-$37^{I20Q/V21S}$ (LLGDFFRKSKEKIGKEFKRQSQRIKDFLRNLVPRTES)	AnHui JYHX CO., LTD	This work
LL-$37^{I20M/V21R}$ (LLGDFFRKSKEKIGKEFKRMRQRIKDFLRNLVPRTES)	AnHui JYHX CO., LTD	This work
Scrm (GLKLRFEFSKIKGEFLKTPEVRFRDIKLKDNRISVQR)	AnHui JYHX CO., LTD	(*15*)
LL20 (LLGDFFRKSKEKIGKEFKRI)	AnHui JYHX CO., LTD	This work
VQ17 (VQRIKDFLRNLVPRTES)	AnHui JYHX CO., LTD	This work
CRAMP (GLLRKGGEKIGEKLKKIGQKIKNFFQKLVPQPEQ)	AnHui JYHX CO., LTD	(*15*)
N-terminally Fluorescein isothiocyanate (FITC)-labeled LL-37	AnHui JYHX CO., LTD	This work
**Antibodies**		
anti-LL-37 antibody	Santa Cruz Biotechnology	sc-166770
anti-GAPDH antibody	Sangon Biotech	D190090
mouse anti-HtrA antiserum	This work	N/A
mouse anti-RpoA antiserum	(*64*)	N/A
**Cell**		
Caco-2	ATCC	HTB-37

Table S18. Primers used in this study.

Primers	Sequence (5'-3')
For strain construction	
pMD19T-*htrA*-up-F	AGAGGATCTACTAGTCATATGGCCTTGCATTAGCTATCAAAGAATAT
pMD19T-*htrA*-up-R	CCTGCAGAAATAACGGAAGGATTTTCCTTTCG
htrA-*kan*^{r}-F	CCTTCCGTTATTTCTGCAGGATAATGCTAAGACAATCA
htrA-*kan*^{r}-R	AGGAAATGAACTGCAGCGCTTATCAATATATCTATAGA
pMD19T-*htrA*-down-F	AGCGCTGCAGTTCATTTCCTTTTGTTCTTAAAGATAAAT
pMD19T-*htrA*-down-R	ACGGCCAGTGAATTCGAGCTCCCTTCTTTGAGTGTTTTTATATTTATTTTTC
pUOA18-P*metK*-*htrA*-F	TTTTAAATGAAAGGAGGATCCATGAAAAAGATTTTTTTATCATTAAGTTTAGC
pUOA18-P*metK*-*htrA*-R	ACGGCCAGTGAATTCGAGCTCTTATTTAAGCAGAAGCAAAGTCGC
pMD19T-*nssR*-up-F	AGAGGATCTACTAGTCATATGGGCATAACACGCACAAATTCTAA
pMD19T-*nssR*-up-R	GTCTTAGCATTATCCTGCAGAATTTAAGGTAGAGAATGGATTTTGATT
nssR-*kan*^{r}-F	TAAATTCTGCAGGATAATGCTAAGACAATCA
nssR-*kan*^{r}-R	TATATACTGCAGCGCTTATCAATATATCTATAGA
pMD19T-*nssR*-down-F	GATAAGCGCTGCAGTATATAATATTTCCATTCTTAACTTATGTTAAATTT
pMD19T-*nssR*-down-R	ACGGCCAGTGAATTCGAGCTCGGAAAAGATAAACAAAAAGATGAAATTT
pUOA18-P*metK*-*nssR*-F	TTTTAAATGAAAGGAGGATCCATGAAAGATTATTTAGAACTTTTATCTAGTGTAGG
pUOA18-P*metK*-*nssR*-R	TTTTAAATGAAAGGAGGATCCATGAAAGATTATTTAGAACTTTTATCTAGTGTAGG
pCold I-*htrA*-F	CTCGGTACCCTCGAGGGATCCATGGCAAGTATTAATTTTAACGAATCG
pCold I-*htrA*-R	CTATCTAGACTGCAGGTCGACTTATTTAAGCAGAAGCAAAGTCGC
pCold I-*HtrA*^{S225A}-F1	CTCGGTACCCTCGAGGGATCCATGGCAAGTATTAATTTTAACGAATCG
pCold I-*HtrA*^{S225A}-R1	ATCCACCAAAGCTCCACCGGCATTTCCTGGATTGATAGAAG
pCold I-*HtrA*^{S225A}-F2	CTTCTATCAATCCAGGAAATGCCGGTGGAGCTTTGGTGGAT
pCold I-*HtrA*^{S225A}-R2	CTATCTAGACTGCAGGTCGACTTATTTAAGCAGAAGCAAAGTCGC
pCold I-*htrA* (81-176)-F	CTCGGTACCCTCGAGGGATCCATGGCAAGTATTAATTTTAACGAATCG
pCold I-*htrA* (81-176)-R	CTATCTAGACTGCAGGTCGACTTATTTAAGCACAAGCAAAGTCGC
pCold I-*nssR*-F	CTCGGTACCCTCGAGGGATCCATGAAAGATTATTTAGAACTTTTATCTAGTGTAGG
pCold I-*nssR*-F	CTATCTAGACTGCAGGTCGACTTACCAAAGTTCTTTCATGATCATATCTT
For DNA pull-down	
Biotin-P*htrA*-F	ATTATAACATAAACTTTAGCCTAAG
P*htrA*-R	AAAGCACTTGCTAAACTTAATGATA
Biotin-P*glyA*-F	GTCTTGTTGTGGCAGGTGAG
P*glyA*-R	ACATGCATCAAAGGACCACC
For EMSA	
FAM-P*htrA*-F	ATTATAACATAAACTTTAGCCTAAG
P*htrA*-F	ATTATAACATAAACTTTAGCCTAAG
P*htrA*-R	AAAGCACTTGCTAAACTTAATGATA
FAM-P*htrA*-1-F	ATTATAACATAAACTTTAGCCTAAG
P*htrA*-1-F	ATTATAACATAAACTTTAGCCTAAG
P*htrA*-1-R	TCATAGTATAAAACCGAAAATTTAC
FAM-P*htrA*-2-F	CACTCAAGGTTTTATTTACAAT
P*htrA*-2-F	CACTCAAGGTTTTATTTACAAT
P*htrA*-2-R	TTTCCTTTTGTTCTTAAAGATAAAT
FAM-P*htrA*-3-F	AGACAGTAAAATATTTTACGAGATA
P*htrA*-3-F	AGACAGTAAAATATTTTACGAGATA
P*htrA*-3-R	AAAGCACTTGCTAAACTTAATGATA
FAM-NssR-b1-F	TTATAACATAAACTTTAGC
NssR-b1-F	TTATAACATAAACTTTAGC
NssR-b1-R	GCTAAAGTTTATGTTATAA
FAM-NssR-b2-F	TTTTATCATTAAGTTTAGC
NssR-b2-F	TTTTATCATTAAGTTTAGC
NssR-b2-R	GCTAAACTTAATGATAAAA
For qRT-PCR	
htrA-RT-F	ATCGACTGCAACGGCTAATC
htrA-RT-R	ATCCAAAGGGCTTGGACG
nssR-RT-F	TATGAAGGCGAAGAGGCTAA
nssR-RT-R	AAAGCAGGCATTTCTGCTATA

glyA (P116B) RT-F	ACATAAAACTTTGCGTGGCCC
glyA (P116B) RT-R	ATGCATCAAAGGACCACCTTGA
CJJ81176_RS03855 RT-F	AGTTCGCTTGTGCCTGTT
CJJ81176_RS03855 RT-R	CCTTAGCCGCACTTCTTAC
CJJ81176_RS01000 RT-F	CGCTAGGCGTTTGGTGA
CJJ81176_RS01000 RT-R	CTACGCATTGGGCTAGGAT
CJJ81176_RS01770 RT-F	ATGCTAAAAGGTATAACCGAACG
CJJ81176_RS01770 RT-R	TTTTGTATCTAGTTGAGGTGGAGT
CJJ81176_RS07420 RT-F	TGCTATTGGTTTTCAAGGATTG
CJJ81176_RS07420 RT-R	CAAGTTCCCTTATTGCGATTT
CJJ81176_RS02340 RT-F	TCCGGTAAAACAGGTCTAG
CJJ81176_RS02340 RT-R	GCAACTTCGCCTTCAATA
CJJ81176_RS07095 RT-F	AGTTAAAAGACGACCAAAGC
CJJ81176_RS07095 RT-R	CAAAGATAACAAAGCCAAAC
CJJ81176_RS07090 RT-F	TGTCCACCAAGCCAAGT
CJJ81176_RS07090 RT-R	ACATACGCAACAGGTAAGAG
CJJ81176_RS03445 RT-F	AACTTGGTAAAACTGTGGGT
CJJ81176_RS03445 RT-R	GTCTGCTTTTCTCGATACTCT
CJJ81176_RS07610 RT-F	AAAAGATGTGCGACAAGTG
CJJ81176_RS07610 RT-R	GCAAATAATGCGAACCAC
CJJ81176_RS05680 RT-F	TTCAACAGCGGTTTCAAATAC
CJJ81176_RS05680 RT-R	TTTAGCCATAGATAAACGACCA
CJJ81176_RS03850 RT-F	TAGAGTGCGAGTGCGTGATC
CJJ81176_RS03850 RT-R	GCTTTTGGCATAAAGTTTGGT
CJJ81176_RS04335 RT-F	TGTTGCTGTTAAGAGTGAAGAAGA
CJJ81176_RS04335 RT-R	CATTACCATGAGGACGATGTG
glyA (81-176) RT-F	ACATAAAACTTTGCGTGGCCC
glyA (81-176) RT-R	TGCATCAAAGGACCACCCTG

Data S1 (separate file). Raw experimental data.

Movie S1 (separate file). Movies of the *C. jejuni* cell treatment with LL-37 (MIC) beginning at $t = 0$. Movie duration is 1.97 min, with images acquired at a rate of one frame per 1 s with exposure time of 30 ms. Left: Phase contrast. Middle: FITC-labeled-LL-37. Right: Sytox Orange.
